# Taxonomic circumscription of melanconis-like fungi causing canker disease in China

**DOI:** 10.3897/mycokeys.42.29634

**Published:** 2018-11-30

**Authors:** Xinlei Fan, Zhuo Du, Jadson D.P. Bezerra, Chengming Tian

**Affiliations:** 1 The Key Laboratory for Silviculture and Conservation of the Ministry of Education, Beijing Forestry University, Beijing 100083, P.R. China Beijing Forestry University Beijing China; 2 State Key Laboratory of Mycology, Institute of Microbiology, Chinese Academy of Sciences, Beijing 100101, P.R. China Institute of Microbiology, Chinese Academy of Sciences Beijing China; 3 Departamento de Micologia Prof. Chaves Batista, Universidade Federal de Pernambuco, Av. Prof. Moraes Rego, s/n, Centro de Biociências, Cidade Universitária, CEP: 50670-901, Recife, PE, Brazil Universidade Federal de Pernambuco Recife Brazil

**Keywords:** Diaporthales, phylogeny, taxonomy, wood-inhabiting fungi

## Abstract

Melanconis-like species comprise latent fungal pathogens with a wide range of woody hosts. Taxonomy of these pathogens is difficult due to their uninformative descriptions and similar asexual morphology. Based on molecular phylogenies, many species of this group were placed in various families of Diaporthales. In this study, eight species of melanconis-like fungi were isolated from *Betulaalbosinensis*, *B.platyphylla* (Betulaceae), *Cornuscontroversa* (Cornaceae), *Corylusmandshurica* (Betulaceae) and *Juglansregia* (Juglandaceae) in China. These species were phylogenetically placed in three families of Diaporhthales, i.e. *Juglanconisjuglandina*, *J.oblonga* (Juglanconidaceae), *Melanconiellabetulicola***sp. nov.**, *M.corylina***sp. nov.** (Melanconiellaceae), *Melanconisbetulae*, *Ms.itoana*, *Ms.stilbostoma* (Melanconidaceae) and one new genus, *Sheathospora* (Melanconiellaceae). *Sheathospora* is proposed to accommodate *Melanconiellacornuta* with conical and discrete pycnidia with aseptate, hyaline, cylindrical to ellipsoidal conidia with distinct hyaline sheath on branches of *Cornuscontroversa*. Combined analyses of ITS, LSU, CAL, RPB2 and TEF1-α sequence data were used to construct the molecular phylogeny. Additionally, we provided separate phylogenetic trees for three families (Juglanconidaceae, Melanconidaceae and Melanconiellaceae) to show the species distribution of melanconis-like fungi in China.

## Introduction

*Melanconium* (Diaporthales) was introduced by Link (1809) from dead branches of *Fagus* with *M.atrum* Link as the generic type. Corda (1837) extended this genus to 28 species. Subsequently, the genera *Melanconis* Tul. & C. Tul. and *Melanconiella* Sacc. were described as sexual morphs of *Melanconium* (Wehmeyer 1937, 1941). Sutton (1980) summarised more than 200 binomials that have been described in *Melanconium*, whereas no generic revision is available due to the uninformative descriptions and illustrations, few morphological characteristics, misplacement or poor condition of original specimens and lacking of ex-type cultures. In the Index Fungorum (2018), there are more than 235 species epithets of *Melanconium* with an estimated 50 species epithets by Kirk et al. (2008). Thus *Melanconium* species has serious obstacles for appropriate interpretation and is phylogenetically distributed throughout the Diaporthales, especially in Juglanconidaceae, Melanconidaceae and Melanconiellaceae. Although the genus *Melanconium* may be synonymous with *Melanconis* and would therefore have priority, the true identity of the generic type, *M.atrum*, is obscure and it was recommended to protect *Melanconis* over *Melanconium* (Rossman et al. 2015).

Molecular phylogenetics have had a major impact in taxonomic rearrangements of fungi since the early 1990s (White et al. 1990, Hibbett et al. 2007, Choi and Kim 2017, Fan et al. 2018). Castlebury et al. (2002) re-evaluated Diaporthales based on LSU rDNA sequences, indicating the single genus *Melanconis* with asexual morph *Melanconium* in Melanconidaceae*s. str.*Rossman et al. (2007) followed this differentiation and believed that many additional species of *Melanconissensu*Wehmeyer (1941) should be separated from Melanconidaceae. One example is *Melanconiellaspodiaea* (Tul. & C. Tul.) Sacc., type of the genus *Melanconiella*, which segregated from *Melanconis* (Rossman et al. 2007). Voglmayr et al. (2012) published sequences and molecular phylogenies for species of *Melanconiella* firstly and proposed that *Melanconiella* represented a distinct clade from *Melanconis*. Subsequently, Norphanphoun et al. (2016) introduced Lamproconiaceae to accommodate *Melanconiumdesmazieri* (Berk. & Broome) Sacc., with its sexual morph *Melanconisdesmazieri* Petr. (Grove 1937, Sutton 1980). Voglmayr et al. (2017) proposed Juglanconidaceae to accommodate *Melanconiumjuglandinum* Kunze. Senanayake et al. (2017) introduced Melanconiellaceae to accommodate the previous unresolved *Melanconiella*.

During trips to collect forest pathogens that cause canker or dieback diseases in China, several melanconis-like taxa associated with various disease symptoms were collected in Beijing, Gansu, Heilongjian, Jilin, Ningxia, Shaanxi and Tibet Provinces. As the higher-level phylogeny of many genera within the melanconis-like taxa remains largely unresolved in China, this project was initiated to address this issue. In this paper, we identified eight melanconis-like species residing in three families of Diaporthales; recognised three genera within Melanoconiellaceae; and described two new species in *Melanconiella* as well as one new genus to accommodate *Melanconiellacornuta*.

## Materials and methods

### Isolation

Fresh specimens of melanconis-like fungi were collected from infected branches of seven hosts during collection trips in China (Table 1). A total of 47 isolates were established by removing a mucoid spore mass from ascomata or conidiomata, spreading the suspension on the surface of 1.8% potato dextrose agar (PDA) and incubating at 25 °C for up to 24 h. Single germinating conidia/ascospores were removed and plated on to fresh PDA plates. Specimens and isolates were deposited in the Key Laboratory for Silviculture and Conservation of the Ministry of Education in the Beijing Forestry University (BJFU) and the working Collection of X.L. Fan (CF) housed at the BJFU. Axenic cultures are maintained in the China Forestry Culture Collection Centre (CFCC).

**Table 1.** Details of the strains included for molecular study used in this study.

**Species**	**Culture/strain/specimen**	**Location**	**Host**	**GenBank accession numbers**				
				**ITS**	**LSU**	**CAL**	**RPB2**	**TEF1-α**
* Apiosporopsis carpinea *	CBS 771.79	Switzerland	* Carpinus betulus *	NA	AF277130	NA	NA	NA
*Apiosporopsis* sp.	11Af2-1	Japan	* Alnus firma *	NA	AB669034	NA	NA	NA
*** Apoharknessia insueta ***	**CBS 111377**	Brazil	* Eucalyptus pellita *	JQ706083	AY720814	NA	NA	NA
	**CBS 11457**5	Colombia	*Eucalyptus* sp.	NA	AY720813	NA	NA	NA
* Asterosporium asterospermum *	MFLU 15-3555	Italy	* Fagus sylvatica *	NA	MF190062	NA	MF377615	NA
	CBS 112404	Italy	* Fagus sylvatica *	NA	AB553745	NA	NA	NA
	KT2138	Japan	* Fagus crenata *	NA	AB553744	NA	NA	NA
*** Auratiopycnidiella tristaniopsidis ***	**CBS 132180 = CPC 16371**	Australia	* Tristaniopsis laurina *	JQ685516	JQ685522	NA	NA	NA
*** Cainiella johansonii ***	**Kruys 731**	Sweden	* Dryas octopetala *	NA	JF701920	NA	NA	NA
*** Chapeckia nigrospora ***	**AR 3809**	USA	*Betula* sp.	JF681957	EU683068	NA	NA	NA
*** Chiangraiomyces bauhiniae ***	**MFLUCC 17-1669**	Thailand	*Bauhinia* sp.	MF190118	MF190064	NA	MF377604	NA
	**MFLUCC 17-1670**	Thailand	*Bauhinia* sp.	MF190119	MF190065	NA	MF377603	NA
*** Chrysocrypta corymbiae ***	**CBS 132528**	Australia	*Corymbia* sp.	JX069867	JX069851	NA	NA	NA
* Coniella diplodiella *	CBS 111858 = CPC 3708	France	* Vitis vinifera *	AY339323	AY339284	NA	KX833423	KX833603
* Coniella koreana *	CBS 143.97	Korea	NA	KX833584	AF408378	NA	KX833490	KX833684
Coniella musaiensis var. hibisci	AR 3534 = CBS 109757	South Africa	*Hibiscus* sp.	KX833589	AF408337	NA	NA	KX833689
* Coniella straminea *	CBS 149.22 = CPC 3932	USA	*Fragaria* sp.	AY339348	AF362569	NA	KX833506	KX833704
* Coniella wangiensis *	CBS 132530 = CPC 19397	Australia	*Eucalyptus* sp.	JX069873	JX069857	NA	KX833509	KX833705
* Coryneum depressum *	AR 3897	Austria	* Quercus cerris *	NA	EU683074	NA	NA	NA
* Coryneum modonium *	AR 3558	Austria	* Castanea sativa *	NA	EU683073	NA	NA	NA
*** Coryneum umbonatum ***	**AR 3541**	Austria	* Quercus cerris *	NA	EU683072	NA	NA	NA
	**MFLUCC 15-1110**	Italy	*Quercus* sp.	MF190121	MF190067	NA	MF377610	NA
	**MFLUCC 13-0658**	Italy	*Quercus* sp.	MF190120	MF190066	NA	MF377609	NA
* Cryphonectria macrospora *	AR 3444 = CBS 109764	Russia	* Quercus mongolica *	EU199182	AF408340	NA	EU220029	NA
* Cryphonectria nitschkei *	AR 3433 = CBS109776	Russia	* Quercus mongolica *	DQ120761	AF408341	NA	NA	NA
*** Cryphonectria parasitica ***	**ATCC 38755**	USA	* Castanea dentata *	AY141856	EU199123	NA	DQ862017	EU222014
*** Cryptodiaporthe aesculi ***	**AFTOL-ID 1238 = CBS 109765**	Austria	* Aesculus hippocastanum *	DQ323530	AF408342	NA	EU199138	GU354004
	**AR3640 = CBS 121905**	USA	* Aesculus hippocastanum *	EU254994	EU255164	NA	EU219269	DQ313558
	**LCM 447.01**	Germany	* Aesculus hippocastanum *	GU367076	NA	NA	GU367110	GU354002
* Cryptosporella betulae *	AR 3524 = CBS 109763	Austria	* Betula pendula *	EU199180	AF408375	NA	EU199139	EU221884
*** Cryptosporella hypodermia ***	**AR 3552**	Austria	* Ulmus minor *	EU199181	AF408346	NA	EU199140	NA
* Cryptosporella suffusa *	AR 3496 = CBS 109750	Austria	* Alnus incana *	EU199207	AF408376	NA	EU199163	EU221945
* Cytospora cenisia *	AR 3522 = CBS 109752	Austria	* Juniperus communis *	NA	AF408385	NA	NA	NA
*** Cytospora chrysosperma ***	**CFCC 89600**	China	* Sophora japonica *	KR045623	KR045623	NA	KU710951	KU710915
* Cytospora elaeagni *	CFCC 89633	China	* Elaeagnus angustifolia *	KF765677	KF765693	NA	KU710956	KU710919
* Cytospora leucostoma *	CFCC 50468	China	* Betula platyphylla *	KT732949	KT732968	NA	NA	NA
* Cytospora nivea *	AR 3512	Austria	* Salix purpurea *	NA	AF408367	NA	NA	NA
* Cytospora sacculus *	AR 3416 = CBS 109756	Russia	* Quercus mongolica *	NA	AF408386	NA	NA	NA
	AR 3426 = CBS 109777	Austria	* Quercus robur *	NA	AF408387	NA	NA	NA
*** Dendrostoma mali ***	**CFCC 52102**	China	* Malus spectabilis *	MG682072	MG682012	NA	MG682032	MG682052
* Dendrostoma osmanthi *	CFCC 52106	China	* Osmanthus fragrans *	MG682073	MG682013	NA	MG682033	MG682053
	CFCC 52107	China	* Osmanthus fragrans *	MG682074	MG682014	NA	MG682034	MG682054
	CFCC 52108	China	* Osmanthus fragrans *	MG682075	MG682015	NA	MG682035	MG682055
	CFCC 52109	China	* Osmanthus fragrans *	MG682076	MG682016	NA	MG682036	MG682056
* Dendrostoma quercinum *	CFCC 52103	China	* Quercus acutissima *	MG682077	MG682017	NA	MG682037	MG682057
	CFCC 52104	China	* Quercus acutissima *	MG682078	MG682018	NA	MG682038	MG682058
	CFCC 52105	China	* Quercus acutissima *	MG682079	MG682019	NA	MG682039	MG682059
* Diaporthe decedens *	AR 3459 = CBS 109772	Austria	* Corylus avellana *	KC343059	AF408348	NA	NA	NA
* Diaporthe detrusa *	AR 3424 = CBS 109770	Austria	* Berberis vulgaris *	KC343061	AF408349	NA	NA	KC343787
*** Diaporthe eres ***	**AR 3538 = CBS 109767**	Austria	* Acer campestre *	KC343075	AF408350	NA	NA	KC343801
* Diaporthella corylina *	CBS 121124	China	*Corylus* sp.	KC343004	NA	NA	NA	NA
*Diaporthella* sp.	CN5	Italy	* Corylus avellana *	KP205483	NA	NA	NA	NA
	CN13	Italy	* Corylus avellana *	KP205484	NA	NA	NA	NA
*** Diaporthosporella cercidicola ***	**CFCC 51994**	China	* Cercis chinensis *	KY852492	KY852515	NA	NA	NA
	**CFCC 51995**	China	* Cercis chinensis *	KY852493	KY852516	NA	NA	NA
	**CFCC 51996**	China	* Cercis chinensis *	KY852494	KY852517	NA	NA	NA
*** Diaporthostoma machili ***	**CFCC 52100**	China	* Machilus leptophylla *	MG682080	MG682020	NA	MG682040	MG682060
	**CFCC 52101**	China	* Machilus leptophylla *	MG682081	MG682021	NA	MG682041	MG682061
*** Disculoides eucalypti ***	**CPC 17650**	Australia	*Eucalyptus* sp.	JQ685517	JQ685523	NA	NA	NA
* Disculoides eucalyptorum *	CBS 132184 = CPC 17648	Australia	* Eucalyptus viminalis *	NR120090	JQ685524	NA	NA	NA
*** Ditopella ditopa ***	**AR 3423 = CBS 109748**	Austria	* Alnus glutinosa *	EU199187	EU199126	NA	EU199145	NA
*** Erythrogloeum hymenaeae ***	**CPC 18819**	Brazil	* Hymenaea courbaril *	JQ685519	JQ685525	NA	NA	NA
*** Gnomonia gnomon ***	**CBS 199.53**	Italy	* Corylus avellana *	AY818956	AF408361	NA	EU219295	EU221885
*** Harknessia eucalypti ***	**CBS 342.97**	Australia	* Eucalyptus regnans *	AY720745	AF408363	NA	NA	NA
* Harknessia leucospermi *	CBS 775.97	South Africa	*Leucospermum* sp.	NR137147	AY720824	NA	NA	NA
* Harknessia molokaiensis *	AR 3578 = CBS 109779	USA	* Eucalyptus robusta *	NA	AF408390	NA	NA	NA
* Harknessia syzygii *	CBS 111124 = CPC184	South Africa	* Syzygium cordatum *	AY720738	AY720834	NA	NA	NA
*** Hercospora tiliae ***	**AR 3526**	Austria	* Tilia tomentosa *	NA	AF408365	NA	NA	NA
*** Hyaliappendispora galii ***	**MFLUCC 16-1208**	Italy	*Galium* sp.	MF190149	MF190095	NA	NA	NA
*** Involutscutellula rubra ***	**CBS 192.71**	Japan	* Quercus phillyraeoides *	MG591899	MG591993	NA	MG976476	MG592086
* Juglanconis appendiculata *	D140	Greece	* Juglans nigra *	KY427138	KY427138	NA	KY427188	KY427207
	D96	Austria	* Juglans nigra *	KY427139	KY427139	NA	KY427189	KY427208
	D96A	Austria	* Juglans nigra *	KY427140	KY427140	NA	KY427190	KY427209
	MC	Greece	* Juglans nigra *	KY427141	KY427141	KY427242	KY427191	KY427210
	MC2	Spain	* Juglans nigra *	KY427142	KY427142	KY427243	KY427192	KY427211
	MC4	Spain	* Juglans nigra *	KY427143	KY427143	KY427244	KY427193	KY427212
	ME17	Austria	* Juglans nigra *	KY427144	KY427144	KY427245	KY427194	KY427213
*** Juglanconis juglandina ***	**D142**	Austria	* Juglans nigra *	KY427145	KY427145	NA	KY427195	KY427214
	CFCC 51727*	China	* Juglans nigra *	KY363854	KY363859	MK096394	MK096439	NA
	CFCC 51728*	China	* Juglans nigra *	KY363855	KY363860	MK096395	MK096440	NA
	CFCC 51729*	China	* Juglans nigra *	KY363856	KY363861	MK096396	MK096441	NA
	**MC1**	Austria	* Juglans nigra *	KY427146	NA	KY427246	KY427196	KY427215
	**MC3**	Spain	* Juglans nigra *	KY427147	KY427146	KY427247	KY427197	KY427216
	**ME16**	Austria	* Juglans nigra *	KY427148	KY427147	KY427248	KY427198	KY427217
	**ME22**	Austria	* Juglans nigra *	KY427149	KY427148	KY427249	KY427199	KY427218
	**ME23**	Austria	* Juglans nigra *	KY427150	KY427150	KY427250	KY427200	KY427219
* Juglanconis oblonga *	CFCC 51725*	China	* Juglans nigra *	KY363852	KY363857	MK096392	MK096437	NA
	CFCC 51726*	China	* Juglans nigra *	KY363853	KY363858	MK096393	MK096438	NA
	ME14	USA	* Juglans cinerea *	KY427151	KY427151	KY427251	KY427201	KY427220
	ME15	USA	* Juglans cinerea *	KY427152	KY427152	KY427252	KY427202	KY427221
	ME18	Japan	* Juglans ailanthifolia *	KY427153	KY427153	KY427253	KY427203	KY427222
	ME19	Japan	* Juglans ailanthifolia *	KY427154	KY427154	KY427254	KY427204	KY427223
* Juglanconis pterocaryae *	ME20	Japan	* Pterocarya rhoifolia *	KY427155	KY427155	KY427255	KY427205	KY427224
*** Lamproconium desmazieri ***	**MFLUCC 14-1047**	Russia	* Tilia cordata *	KX430132	KX430133	NA	NA	MF377592
	**MFLUCC 15-0870**	Russia	* Tilia tomentosa *	KX430134	KX430135	NA	MF377605	MF377591
*Lasmenia* sp.	CBS 124123	Puerto Rico	* Nephelium lappaceum *	GU797406	JF838338	NA	NA	NA
	CBS 124124	Puerto Rico	* Nephelium lappaceum *	JF838336	JF838341	NA	NA	NA
*** Luteocirrhus shearii ***	**CBS 130776**	Australia	* Banksia baxteri *	NR120254	NG042770	NA	NA	NA
*** Macrohilum eucalypti ***	**CPC 10945**	New Zealand	*Eucalyptus* sp.	DQ195781	DQ195793	NA	NA	NA
	**CPC 19421**	Australia	* Eucalyptus piperita *	KR873244	KR873275	NA	NA	NA
* Melanconiella betulicola *	CFCC 52482*	China	* Betula albosinensis *	MK096312	MK096352	NA	MK096397	MK096272
	CFCC 52483*	China	* Betula albosinensis *	MK096313	MK096353	NA	MK096398	MK096273
* Melanconiella carpinicola *	MNM	Austria	* Carpinus betulus *	JQ926232	JQ926232	NA	JQ926304	JQ926370
	MNUK	UK	* Carpinus betulus *	JQ926234	JQ926234	NA	JQ926306	JQ926372
	MSMI	Austria	* Carpinus betulus *	JQ926235	JQ926235	NA	JQ926307	JQ926373
* Melanconiella chrysodiscosporina *	MCH	Austria	* Carpinus betulus *	JQ926238	JQ926238	NA	JQ926310	JQ926376
	MEE	Austria	* Carpinus betulus *	JQ926240	JQ926240	NA	JQ926312	JQ926378
	MGG	Austria	* Carpinus betulus *	JQ926242	JQ926242	NA	JQ926314	JQ926380
* Melanconiella chrysomelanconium *	MCM	Austria	* Carpinus betulus *	JQ926247	JQ926247	NA	JQ926319	JQ926385
	MEUK	UK	* Carpinus betulus *	JQ926249	JQ926249	NA	JQ926321	JQ926387
	MGUK	UK	* Carpinus betulus *	JQ926255	JQ926255	NA	JQ926327	JQ926393
* Melanconiella chrysorientalis *	MGB	Croatia	* Carpinus orientalis *	JQ926256	JQ926256	NA	JQ926328	JQ926394
	MGP	Croatia	* Carpinus orientalis *	JQ926257	JQ926257	NA	JQ926329	JQ926395
	MVH	Croatia	* Carpinus orientalis *	JQ926259	JQ926259	NA	JQ926331	JQ926397
* Melanconiella corylina *	CFCC 52484*	China	* Corylus mandshurica *	MK096314	MK096354	NA	MK096399	MK096274
	CFCC 52485*	China	* Corylus mandshurica *	MK096315	MK096355	NA	MK096400	MK096275
* Melanconiella decorahensis *	CBS 159.26	USA	*Betula* sp.	JQ926260	JQ926260	NA	JQ926332	JQ926398
	MD	France	* Betula pendula *	JQ926261	JQ926261	NA	JQ926333	JQ926399
	MED	France	* Betula pendula *	JQ926262	JQ926262	NA	JQ926334	JQ926400
* Melanconiella echinata *	DAOM 121196	USA	* Carpinus caroliniana *	JQ926263	JQ926263	NA	N/A	N/A
* Melanconiella elegans *	AR 3830	USA	* Carpinus caroliniana *	JQ926264	JQ926264	NA	JQ926335	JQ926401
	BPI 843574	USA	* Carpinus caroliniana *	JQ926266	JQ926266	NA	JQ926337	JQ926403
	BPI 872067	USA	* Carpinus caroliniana *	JQ926267	JQ926267	NA	JQ926338	JQ926404
* Melanconiella ellisii *	BPI 843491	USA	* Carpinus caroliniana *	JQ926268	JQ926268	NA	N/A	JQ926405
	BPI 878343	USA	* Carpinus caroliniana *	JQ926271	JQ926271	NA	JQ926339	JQ926406
	BPI 883227	USA	* Carpinus caroliniana *	JQ926269	JQ926269	NA	N/A	N/A
* Melanconiella flavovirens *	MFV1	Austria	* Corylus avellana *	JQ926274	JQ926274	NA	JQ926342	JQ926409
	MFV2	Austria	* Corylus avellana *	JQ926275	JQ926275	NA	JQ926343	JQ926410
	MFV3	Italy	* Corylus avellana *	JQ926276	JQ926276	NA	JQ926344	JQ926411
* Melanconiella hyperopta *	MCHBV	Austria	* Carpinus betulus *	JQ926280	JQ926280	NA	JQ926346	JQ926413
	MCR	Austria	* Carpinus betulus *	JQ926283	JQ926283	NA	JQ926349	JQ926416
	MHG	Switzerland	* Carpinus betulus *	JQ926285	JQ926285	NA	JQ926351	JQ926418
Melanconiella hyperopta var. orientalis	MHP	Croatia	* Carpinus orientalis *	JQ926288	JQ926288	NA	JQ926352	JQ926420
	MHVA	Croatia	* Carpinus orientalis *	JQ926287	JQ926287	NA	JQ926353	JQ926419
	MSK	Croatia	* Carpinus orientalis *	JQ926286	JQ926286	NA	JQ926354	JQ926421
* Melanconiella meridionalis *	MOA	Austria	* Ostrya carpinifolia *	JQ926289	JQ926289	NA	JQ926355	JQ926422
	MOK	Croatia	* Ostrya carpinifolia *	JQ926290	JQ926290	NA	JQ926356	JQ926423
	MOM	Austria	* Ostrya carpinifolia *	JQ926291	JQ926291	NA	JQ926357	JQ926424
* Melanconiella ostryae *	CBS 208.38	USA	* Ostrya virginiana *	JQ926297	JQ926297	NA	JQ926363	JQ926430
*** Melanconiella spodiaea ***	**MVS**	Croatia	* Carpinus orientalis *	JQ926299	JQ926299	NA	JQ926365	JQ926432
	**MSH**	Austria	* Carpinus betulus *	JQ926298	JQ926298	NA	JQ926364	JQ926431
	**SPOD**	Croatia	* Carpinus betulus *	JQ926300	JQ926300	NA	JQ926366	JQ926433
* Melanconis alni *	AR 3529	Russia	* Duschekia maximowiczii *	NA	AF362566	NA	NA	NA
	AR 3748	Austria	* Alnus viridis *	EU199195	EU199130	NA	EU199153	NA
	AR 4016 = CBS 121480	Austria	* Alnus alnobetula *	EU254863	NA	NA	EU219298	EU221894
	CBS 109773	Austria	* Alnus viridis *	DQ323523	AF408371	NA	EU219300	EU221896
* Melanconis betulae *	CFCC 50471*	China	* Betula albosinensis *	KT732952	KT732971	NA	KT732984	KT733001
	CFCC 50472*	China	* Betula albosinensis *	KT732953	KT732972	NA	KT732985	KT733002
	CFCC 50473*	China	* Betula albosinensis *	KT732954	KT732973	NA	KT732986	KT733003
* Melanconis italica *	MFLUCC 16-1199	Italy	* Alnus cordata *	MF190151	MF190096	NA	NA	NA
	MFLUCC 17-1659	Italy	* Alnus cordata *	MF190151	MF190097	NA	MF377602	NA
* Melanconis itoana *	CFCC 50474*	China	* Betula albosinensis *	KT732955	KT732974	NA	KT732987	KT733004
	CFCC 52876*	China	* Betula albosinensis *	MK096324	MK096364	NA	MK096409	MK096284
	CFCC 52877*	China	* Betula albosinensis *	MK096326	MK096366	NA	MK096411	MK096286
	CFCC 52878*	China	* Betula albosinensis *	MK096327	MK096367	NA	MK096412	MK096287
	MAFF 410080	Japan	* Betula ermanii *	JX522738	NA	NA	NA	NA
* Melanconis marginalis *	AR 3442 = CBS 109744	Canada	* Alnus rubra *	EU199197	AF408373	NA	EU219301	EU221991
	MAFF 410218	Japan	* Alnus maximowiczii *	JX522742	NA	NA	NA	NA
*** Melanconis stilbostoma ***	**CBS 109778 = AR 3501**	Austria	* Betula pendula *	DQ323524	AF408374	NA	EU219299	EU221886
	**CBS 121894 = MS**	NA	* Betula pendula *	JQ926229	JQ926229	NA	JQ926302	JQ926368
	CFCC 50475*	China	* Betula platyphylla *	KT732956	KT732975	NA	KT732988	KT733005
	CFCC 50476*	China	* Betula platyphylla *	KT732957	KT732976	NA	KT732989	KT733006
	CFCC 50477*	China	* Betula platyphylla *	KT732958	KT732977	NA	KT732990	KT733007
	CFCC 50478*	China	* Betula platyphylla *	KT732959	KT732978	NA	KT732991	KT733008
	CFCC 50479*	China	* Betula platyphylla *	KT732960	KT732979	NA	KT732992	KT733009
	CFCC 50480*	China	* Betula platyphylla *	KT732961	KT732980	NA	KT732993	KT733010
*** Melanconis stilbostoma ***	CFCC 50481*	China	* Betula platyphylla *	KT732962	KT732981	NA	KT732994	KT733011
	CFCC 50482*	China	* Betula platyphylla *	KT732963	KT732982	NA	KT732995	KT733012
	CFCC 52843*	China	* Betula platyphylla *	MK096338	MK096378	NA	MK096423	MK096298
	CFCC 52844*	China	* Betula platyphylla *	MK096341	MK096381	NA	MK096426	MK096301
	CFCC 52845*	China	* Betula platyphylla *	MK096343	MK096383	NA	MK096428	MK096303
	CFCC 52846*	China	* Betula platyphylla *	MK096347	MK096387	NA	MK096432	MK096307
	CFCC 52847*	China	* Betula platyphylla *	MK096348	MK096388	NA	MK096433	MK096308
	CFCC 52848*	China	* Betula platyphylla *	MK096349	MK096389	NA	MK096434	MK096309
	CFCC 52849*	China	* Betula platyphylla *	MK096328	MK096368	NA	MK096413	MK096288
	CFCC 52850*	China	* Betula platyphylla *	MK096329	MK096369	NA	MK096414	MK096289
	CFCC 52851*	China	* Betula platyphylla *	MK096330	MK096370	NA	MK096415	MK096290
	CFCC 52852*	China	* Betula platyphylla *	MK096331	MK096371	NA	MK096416	MK096291
	CFCC 52853*	China	* Betula platyphylla *	MK096332	MK096372	NA	MK096417	MK096292
	CFCC 52854*	China	* Betula platyphylla *	MK096333	MK096373	NA	MK096418	MK096293
	CFCC 52855*	China	* Betula platyphylla *	MK096334	MK096374	NA	MK096419	MK096294
	CFCC 52856*	China	* Betula platyphylla *	MK096335	MK096375	NA	MK096420	MK096295
	CFCC 52857*	China	* Betula platyphylla *	MK096336	MK096376	NA	MK096421	MK096296
	CFCC 52858*	China	* Betula platyphylla *	MK096337	MK096377	NA	MK096422	MK096297
	CFCC 52859*	China	* Betula platyphylla *	MK096339	MK096379	NA	MK096424	MK096299
	CFCC 52860*	China	* Betula platyphylla *	MK096340	MK096380	NA	MK096425	MK096300
	CFCC 52861*	China	* Betula platyphylla *	MK096342	MK096382	NA	MK096427	MK096302
	CFCC 52862*	China	* Betula platyphylla *	MK096344	MK096384	NA	MK096429	MK096304
	CFCC 52863*	China	* Betula platyphylla *	MK096345	MK096385	NA	MK096430	MK096305
	CFCC 52864*	China	* Betula platyphylla *	MK096346	MK096386	NA	MK096431	MK096306
	CFCC 52865*	China	* Betula platyphylla *	MK096316	MK096356	NA	MK096401	MK096276
	CFCC 52866*	China	* Betula platyphylla *	MK096317	MK096357	NA	MK096402	MK096277
	CFCC 52867*	China	* Betula platyphylla *	MK096318	MK096358	NA	MK096403	MK096278
	CFCC 52868*	China	* Betula platyphylla *	MK096319	MK096359	NA	MK096404	MK096279
	CFCC 52869*	China	* Betula platyphylla *	MK096320	MK096360	NA	MK096405	MK096280
	CFCC 52870*	China	* Betula platyphylla *	MK096321	MK096361	NA	MK096406	MK096281
	CFCC 52871*	China	* Betula platyphylla *	MK096322	MK096362	NA	MK096407	MK096282
	CFCC 52872*	China	* Betula platyphylla *	MK096323	MK096363	NA	MK096408	MK096283
	CFCC 52873*	China	* Betula platyphylla *	MK096350	MK096390	NA	MK096435	MK096310
	CFCC 52874*	China	* Betula platyphylla *	MK096351	MK096391	NA	MK096436	MK096311
	CFCC 52875*	China	* Betula platyphylla *	MK096325	MK096365	NA	MK096410	MK096285
* Microascospora fragariae *	CBS 118.16	USA	*Fragaria* sp.	NR156500	NA	NA	NA	NA
	CBS 128350	USA	*Rubus* sp.	JF514854	NA	NA	NA	NA
	1-1	China	* Fragaria ananassa *	HM854850	NA	NA	NA	NA
	1-2	China	* Fragaria ananassa *	HM854849	NA	NA	NA	NA
	1-3	China	* Fragaria ananassa *	HM854852	NA	NA	NA	NA
*** Microascospora rubi ***	**MFLU 15-1112**	Italy	* Rubus ulmifolia *	MF190154	MF190098	NA	MF377581	MF377611
	**MFLU 17-0883**	Italy	* Rubus ulmifolia *	MF190153	MF190099	NA	MF377582	MF377612
*** Nakataea oryzae ***	**CBS 243.76**	NA	NA	KM484861	DQ341498	NA	NA	NA
*** Oblongisporothyrium castanopsidis ***	**ATCC 22470**	Japan	* Castanopsis cuspidata *	MG591850	MG591943	NA	MG592038	MG976454
*** Ophiodiaporthe cyatheae ***	**YMJ1364**	China	* Cyathea lepifera *	JX570889	JX570891	NA	JX570893	NA
*** Pachytrype princeps ***	**Rogers S**	USA	NA	NA	FJ532382	NA	NA	NA
* Pachytrype rimosa *	FF1066	Costa Rica	NA	NA	FJ532381	NA	NA	NA
*** Paradiaporthe artemisiae ***	**MFLUCC 14-0850**	Italy	*Artemisia* sp.	MF190155	MF190100	NA	NA	NA
	**MFLUCC 17-1663**	Italy	*Artemisia* sp.	MF190156	MF190101	NA	NA	NA
*** Phaeoappendispora thailandensis ***	**MFLUCC 13-0161**	Thailand	*Quercus* sp.	MF190157	MF190102	NA	MF377613	NA
*** Phaeodiaporthe appendiculata ***	**CBS 123821 = D77**	Austria	* Acer campestre *	KF570156	KF570156	NA	NA	NA
	**CBS 123809 = D76**	Austria	* Acer campestre *	KF570155	KF570155	NA	NA	NA
*** Phragmoporthe conformis ***	**AR 3632 = CBS 109783**	Canada	* Alnus rubra *	DQ323527	AF408377	NA	NA	NA
*** Plagiostoma euphorbiae ***	**CBS 340.78**	Netherlands	* Euphorbia palustris *	EU199198	AF408382	NA	DQ368643	NA
* Plagiostoma salicellum *	AR 3455 = CBS 109775	Austria	*Salix* sp.	DQ323529	AF408345	NA	EU199141	EU221916
*** Prosopidicola mexicana ***	**CBS 113530**	USA	* Prosopis glandulosa *	AY720710	NA	NA	NA	NA
	**CBS 113529**	USA	* Prosopis glandulosa *	AY720709	KX228354	NA	NA	NA
*** Pseudomelanconis caryae ***	**CFCC 52110**	China	* Carya cathayensis *	MG682082	MG682022	NA	MG682042	MG682062
	**CFCC 52111**	China	* Carya cathayensis *	MG682083	MG682023	NA	MG682043	MG682063
	**CFCC 52112**	China	* Carya cathayensis *	MG682084	MG682024	NA	MG682044	MG682064
	**CFCC 52113**	China	* Carya cathayensis *	MG682085	MG682025	NA	MG682045	MG682065
*** Pseudoplagiostoma eucalypti ***	**CBS 124807**	Venezuela	* Eucalyptus urophylla *	GU973512	GU973606	NA	NA	NA
	**CBS 116382**	Thailand	* Eucalyptus camaldulensis *	GU973514	GU973608	NA	NA	NA
* Pseudoplagiostoma oldii *	CBS 115722	Australia	* Eucalyptus camaldulensis *	GU973535	GU973610	NA	NA	NA
* Pseudoplagiostoma variabile *	CBS 113067	Uruguay	* Eucalyptus globulus *	GU973536	GU973611	NA	NA	NA
*** Pyricularia grisea ***	**Ina168**	NA	NA	AB026819	AB026819	NA	NA	NA
* Racheliella saprophytica *	NTCL052-1	Thailand	* Syzygium cumini *	KJ021933	KJ021935	NA	NA	NA
*** Racheliella wingfieldiana ***	**CBS 143669**	South Africa	* Syzigium guineense *	MG591911	MG592006	NA	MG592100	MG976487
*** Rossmania ukurunduensis ***	**AR 3484**	Russia	* Acer ukurunduense *	NA	EU683075	NA	NA	NA
*** Saprothyrium thailandense ***	**MFLU 13-0260**	Thailand	Decaying leaf	MF190163	MF190110	NA	NA	NA
*** Sheathospora cornuta ***	CFCC 51990*	China	* Cornus controversa *	MF360006	MF360008	NA	MF360002	MF360004
	CFCC 51991*	China	* Juglans regia *	MF360007	MF360009	NA	MF360003	MF360005
*** Sillia ferruginea ***	**AR 3440 = CBS 126567**	Austria	* Corylus avellana *	JF681959	EU683076	NA	NA	NA
*** Sphaerosporithyrium mexicanum ***	**CFNL 2945**	Mexico	* Quercus eduardi *	MG591896	MG591990	NA	MG592083	MG976473
* Stegonsporium protopyriforme *	CBS 117041	Austria	* Acer pseudoplatanus *	NR126119	EU039992	NA	NA	NA
*** Stegonsporium pyriforme ***	**CBS 124487**	UK	* Acer heldreichii *	KF570160	KF570160	NA	KF570190	NA
*** Stilbospora macrosperma ***	**CBS 121883**	Austria	* Carpinus betulus *	JX517290	JX517299	NA	KF570196	NA
	**CBS 121695**	Netherlands	* Carpinus betulus *	JX517288	JX517297	NA	NA	NA
* Sydowiella depressula *	CBS 813.79	Switzerland	*Rubus* sp.	NA	EU683077	NA	NA	NA
*** Sydowiella fenestrans ***	**AR 3777 = CBS 125530**	Russia	* Chamerion angustifolium *	JF681956	EU683078	NA	NA	NA
* Synnemasporella aculeans *	AR 3878 = CBS 126566	USA	* Rhus glabra *	NA	EU255134	NA	NA	NA
	CFCC 52094	China	* Rhus chinensis *	MG682086	MG682026	NA	MG682046	MG682066
	CFCC 52095	China	* Rhus chinensis *	MG682087	MG682027	NA	MG682047	MG682067
	CFCC 52096	China	* Rhus chinensis *	MG682088	MG682028	NA	MG682048	MG682068
*** Synnemasporella toxicodendri ***	**CFCC 52097**	China	* Toxicodendron sylvestre *	MG682089	MG682029	NA	MG682049	MG682069
	**CFCC 52098**	China	* Toxicodendron sylvestre *	MG682090	MG682030	NA	MG682050	MG682070
	**CFCC 52099**	China	* Toxicodendron sylvestre *	MG682091	MG682031	NA	MG682051	MG682071
*** Tubakia japonica ***	**ATCC 22472**	Japan	* Castanea crenata *	MG591886	MG591978	NA	MG592071	MG976465
	**CBS 191.71**	Japan	* Castanea crenata *	MG591885	MG591977	NA	MG592070	MG976464
	**MUCC 2297**	Japan	* Castanea crenata *	NA	MG591979	NA	MG592072	MG976466
	**MUCC 2298**	Japan	* Castanea crenata *	NA	MG591980	NA	MG592073	MG976467
	**MUCC 2300**	Japan	* Castanea crenata *	NA	MG591981	NA	MG592074	MG976468
	**MUCC 2301**	Japan	* Castanea crenata *	NA	MG591982	NA	MG592075	MG976469
* Tubakia seoraksanensis *	CBS 127490	South Korea	* Quercus mongolica *	MG591907	KP260499	NA	MG592094	NA
* Tubakia sutoniana *	ICMP 14042	New Zealand	*Quercus* sp.	KC145909	NA	NA	NA	KC145954
	ICMP 14043	New Zealand	* Quercus ilex *	KC145858	NA	NA	NA	KC145955

Note: ATCC: American Type Culture Collecton, Virginia, USA; CBS: Westerdijk Fungal Biodiversity Institute (CBS-KNAW Fungal Biodiversity Centre), Utrecht, The Netherlands; CFCC: China Forestry Culture Collection Centre, Beijing, China; CFNL: Herbarium and culture collection at the Faculty of Forestry Sciences, University of Nuevo León, México; CPC: Culture collection of Pedro Crous, The Netherlands; ICMP: International Collection of Microorganisms from Plants, New Zealand; MFLU: Mae Fah Luang University herbarium, Thailand; MFLUCC: Mae Fah Luang University Culture Collection, Thailand; MUCC (Japan): Culture Collection, Laboratory of Plant Pathology, Mie University, Tsu, Mie Prefecture, Japan; NA: not applicable. All the new isolates used in this study are marked by an asterisk (*) and the strains from generic type species are in bold.

### Morphological studies

Species identification was based on morphological features of the ascomata or conidiomata produced on infected plant tissues and micromorphology, supplemented by cultural characteristics. Cross-sections were prepared by hand using a double-edge blade under a dissecting microscope. More than 10 conidiomata/ascomata, 10 asci and/or 50 conidia/ascospores were measured to calculate the mean size and standard deviation (SD). Microscopic photographs were captured with a Nikon Eclipse 80i microscope equipped with a Nikon digital sight DS-Ri2 high definition colour camera, using differential interference contrast (DIC) illumination and the Nikon software NIS-Elements D Package v. 3.00. Adobe Bridge CS v. 6 and Adobe Photoshop CS v. 5 were used for the manual editing. Nomenclatural novelties and descriptions were deposited in MycoBank (Crous et al. 2004). Colony diameters were measured and the colony colours described after 3 weeks according to the colour charts of Rayner (1970).

### DNA extraction and sequencing

Genomic DNA was extracted using a modified CTAB method, with fungal mycelium harvested from PDA plates with cellophane (Doyle and Doyle 1990). The DNA was estimated by electrophoresis in 1% agarose gel and the quality was measured by NanoDrop 2000 (Thermo, USA) according to the user’s manual (Desjardins et al. 2009). The PCR amplifications were performed in DNA Engine (PTC-200) Peltier Thermal Cycler (Bio-Rad Laboratories, CA, USA). The ITS region was amplified with the primers ITS1 and ITS4 (White et al. 1990), the LSU region with the primers LR0R and LR5 (Vilgalys and Hester 1990), the CAL gene (for Juglanconidaceae) with primers CAL-228F and CAL-737R (Carbone and Kohn 1999), the RPB2 region with primers fRPB2-5F and fRPB2-7cR (Liu et al. 1999), the TEF1-α gene with the primers EF1-728F and EF1-LLErev for Melanconiellaceae (Carbone and Kohn 1999, Jaklitsch et al. 2005) and the primers EF1-983F and EF1-1567R for Melanconidaceae (Carbone and Kohn 1999, Rehner and Buckley 2005). The PCR mixture for all the regions consisted of 1 μl genomic DNA, 3 mM MgCl_2_, 20 μM of each dNTP, 0.2 μM of each primer and 0.25 U BIOTAQ DNA polymerase (Bioline). Conditions for PCR of ITS and LSU regions constituted an initial denaturation step of 2 min at 95 °C, followed by 35 cycles of 30 s at 95 °C, 45 s at 51 °C and 1 min at 72 °C and a final extension step of 8 min at 72 °C, while the TEF1-α gene was performed using an initial denaturation step of 2 min at 95 °C, followed by 35 cycles of 30 s at 95 °C, 45 s at 56 °C and 1 min at 72 °C and a final extension step of 8 min at 72 °C. For the RPB2 amplification, conditions consisted of five cycles of 45 s at 95 °C, 45 s at 56 °C and 2 min at 72 °C, then five cycles with a 53 °C annealing temperature and 30 cycles with a 50 °C annealing temperature. The DNA sequencing was performed using an ABI PRISM 3730XL DNA Analyzer with BigDye Terminater Kit v. 3.1 (Invitrogen) at the Shanghai Invitrogen Biological Technology Company Limited (Beijing, China).

### Phylogenetic analyses

DNA sequences generated by each primer combination were used to obtain consensus sequences using SeqMan v. 7.1.0 in the DNASTAR Lasergene Core Suite software package (DNASTAR Inc., Madison, WI, USA). Reference sequences were selected based on ex-type or ex-epitype sequences available from relevant published literature (Voglmayr et al. 2012, 2017, Fan et al. 2016, 2018, Du et al. 2017, Senanayake et al. 2017) (Table 1). All sequences were aligned using MAFFT v. 7 (http://mafft.cbrc.jp/alignment/server/index.html) and edited manually using MEGA v. 6 (Tamura et al. 2013). Phylogenetic analyses were performed using PAUP v. 4.0b10 for maximum parsimony (MP) analysis (Swofford 2003), MrBayes v. 3.1.2 for Bayesian Inference (BI) analysis (Ronquist and Huelsenbeck 2003) and PhyML v. 7.2.8 for Maximum Likelihood (ML) analysis (Guindon et al. 2010). The first analyses were performed on the combined multi-gene dataset (ITS, LSU, RPB2, TEF1-α) to compare isolates of Diaporthales species to ex-type sequence data from recent studies (Table 1).

A partition homogeneity test (PHT) with heuristic search and 1 000 search replicates was performed using PAUP to test for incongruence amongst the ITS, LSU, RPB2 and TEF1-α sequence datasets in reconstructing phylogenetic trees. Maximum parsimony (MP) analysis was run using 1 000 heuristic search replicates with random-additions of sequences with a tree bisection and reconnection (TBR) algorithm. Maxtrees were set to 5 000, branches of zero length were collapsed and all equally parsimonious trees were saved. Other calculated parsimony scores were tree length (TL), consistency index (CI), retention index (RI) and rescaled consistency (RC). Maximum likelihood (ML) analysis was performed with a GTR site substitution model, including a gamma-distributed rate heterogeneity and a proportion of invariant sites (Guindon et al. 2010). The branch support was evaluated with a bootstrapping (BS) method of 1 000 replicates.

MrModeltest v. 2.3 was used to estimate the best nucleotide substitution model settings for each gene (Posada and Crandall 1998). Bayesian inference (BI) was performed based on the DNA dataset from the results of the MrModeltest, using a Markov Chain Monte Carlo (MCMC) algorithm in MrBayes v. 3.1.2 (Ronquist and Huelsenbeck 2003). Two MCMC chains were run from random trees for 1 000 M generations and stopped when the average standard deviation of split frequencies fell below 0.01. Trees were saved each 1 000 generations. The first 25% of trees were discarded as the burn-in phase of each analysis and the posterior probabilities (BPP) were calculated from the remaining trees (Rannala and Yang 1996).

In addition to the above analyses, we provided separate phylogenetic trees for Juglanconidaceae, Melanconidaceae and Melanconiellaceae, based on various gene regions (see below) and the same analyses parameters as given above. Phylograms were edited using FigTree v. 1.3.1 (Rambaut and Drummond 2010). Novel sequences generated in the current study were deposited in GenBank (Table 1). The aligned matrices used for phylogenetic analyses and the resulting trees can be found in TreeBASE (www.treebase.org; accession number: S23477).

## Results

### Phylogenetic analyses

**Figure 1.**
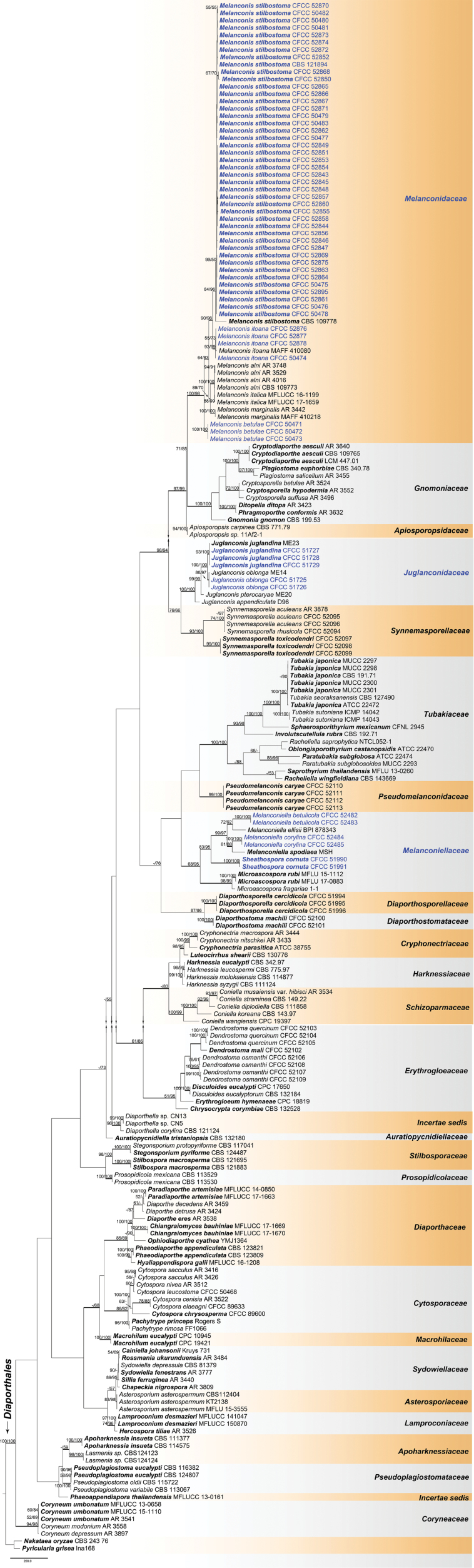
Phylogram of Diaporthales obtained from an MP analysis of a combined matrix of ITS, LSU, RPB2 and TEF1-α. MP and ML bootstrap support values above 50% are shown at the first and second position, respectively. Thickened branches represent posterior probabilities above 0.95 from BI. Scale bar = 200 changes. Type species are in bold. Strains obtained in the current study are in blue.

The combined matrix of ITS, LSU, RPB2 and TEF1-α of Diaporthales included 209 ingroup and two outgroup taxa, comprising 3 269 characters including gaps (776 characters for ITS, 517 for LSU, 1107 for RPB2 and 869 for TEF1-α) in the aligned matrix. Of these, 1 417 characters were constant, 192 variable characters were parsimony-uninformative and 1 660 characters were parsimony informative. The MP analysis resulted in 100 most parsimonious trees (TL = 10 370, CI = 0.341, RI = 0.806, RC = 0.275) and the first tree is shown as Fig. 1. The MP and ML bootstrap support values above 50% are shown at the first and second position, respectively. Branches with significant Bayesian posterior probability (≥ 0.95) in Bayesian analyses were thickened in the phylogenetic tree. The phylogram based on four genes resolved 28 known lineages, representing 26 known families and two *incertae sedis* genera *Diaporthella* and *Phaeoappendispora* due to lack of sequence data on their types. The current 47 melanconis-like isolates are herein placed within Juglanconidaceae, Melanconidaceae and Melanconiellaceae in Diaporthales (Fig. 1). A phylogenetic tree of each family or genus was constructed separately based on different DNA datasets. Tree topologies of all genera computed from the MP, ML and Bayesian analyses were similar for the individual gene region and in the combined dataset.

**Figure 2.**
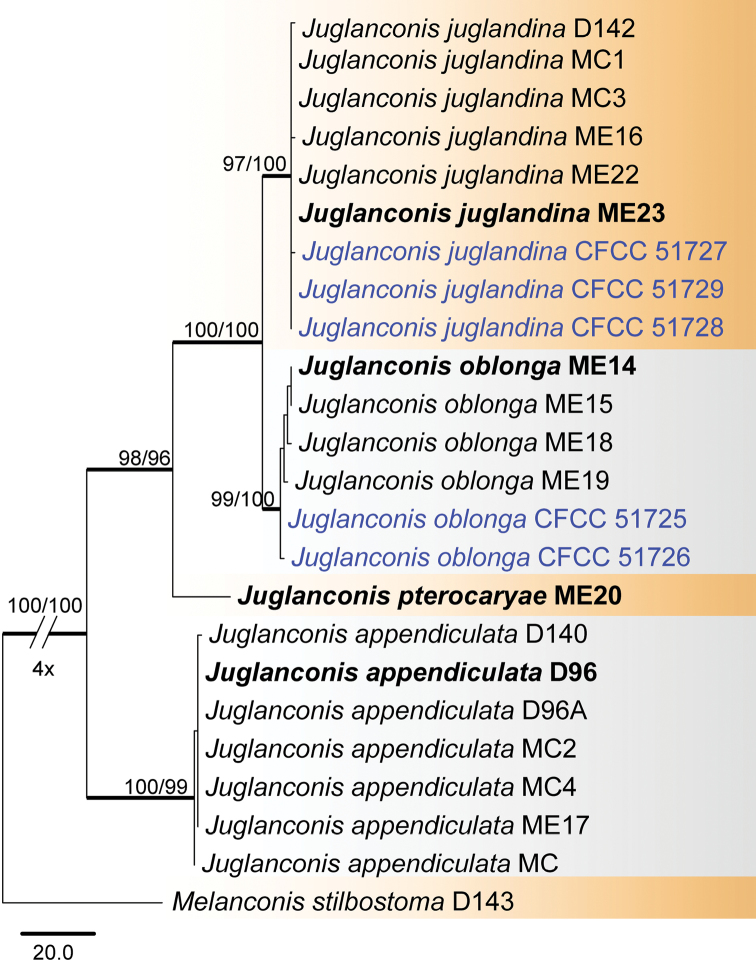
Phylogram of *Juglanconis* (Juglanconidaceae) obtained from an MP analysis of a combined matrix of ITS, LSU, CAL and RPB2. MP and ML bootstrap support values above 50% are shown at the first and second position, respectively. Thickened branches represent posterior probabilities above 0.95 from BI. Scale bar = 20 changes. Type species are in bold. Strains obtained in the current study are in blue.

For the single genus *Juglanconis* (Juglanconidaceae), a combined ITS, LSU, CAL and RPB2 matrix of 23 ingroup accessions (five from this study and 18 retrieved from GenBank) was produced, which comprised 2 736 characters including gaps (2 427 constant, 216 variable and parsimony-uninformative, 93 parsimony-informative). A heuristic MP search generated nine equally most parsimonious trees (TL = 332, CI = 0.976, RI = 0.985, RC = 0.961), one of which is shown in Fig. 2. Isolates of *Juglanconis* clustered in four clades, corresponding to the four known species in this genus. The five Chinese strains sequenced in this study were revealed to belong to *Juglanconisjuglandina* (3) and *J.oblonga* (2).

For Melanconiellaceae, a combined ITS, LSU, RPB2 and TEF1-α matrix was produced from 53 ingroup accessions (six from this study and 47 retrieved from GenBank), which comprised 4 122 characters including gaps (2 829 constant, 87 variable and parsimony-uninformative, 1 206 parsimony-informative). A heuristic MP search generated 24 most parsimonious trees (TL = 2 716, CI = 0.652, RI = 0.880, RC = 0.573), one of which is shown in Fig. 5. Isolates of Melanconiellaceae clustered in three clades, corresponding to the type genus *Melanconiella*, *Microascospora* and a lineage described as the new genus *Sheathospora* below. *Melanconiellabetulicola* and *M.corylina* formed two distinct strongly supported clades (MP/ML/BI = 100/100/1), which differ from the other species of the *Melanconiella* clade.

For the single genus *Melanconis* (Melanconidaceae), a combined ITS, LSU, RPB2 and TEF1-α matrix was produced for 57 ingroup accessions (49 from this study and eight retrieved from GenBank), which comprised 2 597 characters including gaps (2 238 constant, 219 variable and parsimony-uninformative, 140 parsimony-informative). A heuristic MP search generated 144 most parsimonious trees (TL = 459, CI = 0.861, RI = 0.919, RC = 0.791), one of which is shown in Fig. 6. Isolates of *Melanconis* clustered in six clades, corresponding to six known species in this genus. *Melanconisbetulae*, *Ms.stilbostoma* and *Ms.itoana* were confirmed from China in this study.

## Taxonomy

### Juglanconidaceae

Taxon classificationFungiDiaporthalesJuglanconidaceae

Voglmayr & Jaklitsch, Persoonia 38: 142 (2017)

#### Type genus.

*Juglanconis* Voglmayr & Jaklitsch, Persoonia 38: 142 (2017)

#### Notes.

Juglanconidaceae, with the single genus *Juglanconis*, was newly introduced by Voglmayr et al. (2017) for *Melanconiumjuglandinum*, *M.oblongum* and *M.pterocaryae*. In this paper, we provide an updated tree including accessions of two *Juglanconis* species from China (Fig. 2).

### Juglanconis

Taxon classificationFungiDiaporthalesJuglanconidaceae

Voglmayr & Jaklitsch, Persoonia 38: 142 (2017)

#### Type species.

*Juglanconisjuglandina* (Kunze) Voglmayr & Jaklitsch, Persoonia 38: 144 (2017).

#### Notes.

*Juglanconis* was newly introduced by Voglmayr et al. (2017). The genus is characterised by having perithecial ascomata, octosporous asci with an apical ring, hyaline, bicellular ascospores with or without gelatinous appendages and acervular conidiomata with brown conidia with gelatinous sheaths and with verruculous inner surface of the conidal wall (Voglmayr et al. 2017). *Juglanconis* includes four species (*J.appendiculata*, *J.juglandina*, *J.oblonga* and *J.pterocariae*), which were restricted to host in Juglandaceae (Voglmayr et al. 2017).

### Juglanconis juglandina

Taxon classificationFungiDiaporthalesJuglanconidaceae

(Kunze) Voglmayr & Jaklitsch, Persoonia 38: 144 (2017)

Fig. 3

≡Melanconiumjuglandinum Kunze, Fl. Dresd., 2. Aufl.: 260. 1823.

#### Descriptions.

Conidiomata acervular, immersed in host bark, erumpent from surface of host branches, scattered or occasionally confluent, 1.5–2.5 mm, covered by black discharged conidial masses at maturity, usually conspicuous. Ectostromatic disc straw to honey, surrounded by bark or not. Central column beneath the disc more or less conical, straw to buff. Conidiophores cylindrical to lageniform, simple, rarely branched at the base, smooth, subhyaline to pale brown. Conidiogenous cells annellidic with distinct annellations, integrated. Conidia unicellular, initially hyaline, becoming brown to blackish when mature, broadly ellipsoid to broadly pip-shaped, truncate with distinct scar at the base, densely multiguttulate, thick-walled, (17–)19–22(–24.5) × (9–)11–14(–16.5) μm (av. = 20 × 13 μm, n = 50), with 0.8–1 µm wide gelatinous sheath. Sexual morph was not observed.

**Figure 3.**
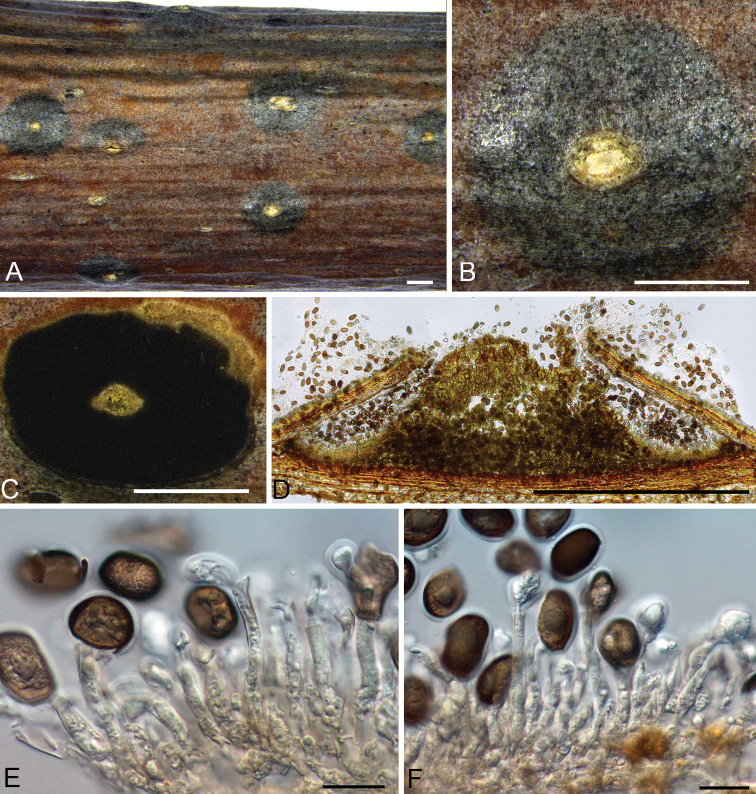
Morphology of *Juglanconisjuglandina* from *Juglansregia*. **A–B** habit of acervuli on branches **C** transverse section through acervulus **D** longitudinal section through acervulus **E–F** conidiophores, conidiogenous cells and conidia. Scale bars: 1 mm (**A–D**), 20 μm (**E–F**).

#### Culture characteristics.

On PDA, cultures are initially white, becoming straw after 3–5 d and grey olivaceous after 7–10 d. The colonies are felty with an irregular edge; sterile.

#### Materials examined.

(all on twigs and branches of *Juglansregia*). CHINA, Gansu Province, Qingyang City, Shishe village, 35°38'17.08"N, 107°47'48.68"E, 14 July 2013, X.L. Fan (BJFC-S908; living culture, CFCC 51727); Gansu Province, Qingyang City, Zhongwan Forest Farm, 35°26'26.33"N, 108°34'09.38"E, 11 July 2013, X.L. Fan (BJFC-S947; living culture, CFCC 51728); Gansu Province, Qingyang City, Zhongwan Forest Farm, 35°26'25.52"N, 108°34'09.03"E, 11 July 2013, X.L. Fan (BJFC-S955; living culture, CFCC 51729).

#### Notes.

*Juglanconisjuglandina* is the type species of *Juglanconis* and is thus far only known to occur on *Juglansregia* distributed in Asia and Europe (Voglmayr et al. 2017). *Juglanconisjuglandina* is described based on *Melanconiumjuglandinum* (= *Melanconiscarthusiana*) (Voglmayr et al. 2017), which was regarded as the main causal agent of canker and dieback disease of *Juglansregia* in China (China Microbiology and Virology Databases, http://www.micro.csdb.cn/).

### Juglanconis oblonga

Taxon classificationFungiDiaporthalesJuglanconidaceae

(Berk.) Voglmayr & Jaklitsch, Persoonia 38: 147 (2017)

Fig. 4

≡ Melanconiumoblongum Berk., Grevillea 2 (no. 22): 153. 1874.
= Diaporthejuglandis Ellis & Everh., Proc. Acad. Nat. Sci. Philadelphia 45: 448. 1893.
≡ Melanconisjuglandis (Ellis & Everh.) A.H. Graves, Phytopathology 13: 311. 1923.

#### Descriptions.

Pseudostromata immersed in host bark, distinctly erumpent from surface of host branches, 1.5–3 mm diam. Ectostromatic disc indistinct, usually circular, greyish to brownish. Perithecia often appearing as rounded bumps beneath the bark surface surrounding the ectostromatic disc, prolonged black neck from the top, (450–)525–700(–780) µm diam. (av. = 580 μm, n = 30). Asci hyaline, clavate to fusoid, (120–)122–135 × (12.5–)13–16.5 (–17) μm (av. = 126.5 × 15 μm, n = 20). Ascospores hyaline, ellipsoid, broadly ellipsoid or broadly fusoid, symmetric to slightly asymmetric, straight, rarely slightly curved, constricted at the septum, (17–)17.5–22(–23.5) × (7.5–)8–10.5(–11) μm (av. = 19.5 × 9.5 μm, n = 50). Conidiomata acervular, immersed in host bark, erumpent from surface of host branches, scattered or occasionally confluent, 1–2 mm, covered by black discharged conidial masses at maturity, usually conspicuous. Ectostromatic disc buff to honey, surrounded by bark or not. Central column beneath the disc more or less conical, isabelline to olivaceous grey. Conidiophores cylindrical to lageniform, simple, rarely branched at the base, smooth, subhyaline to pale brown. Conidiogenous cells annellidic with distinct annellations, integrated. Conidia unicellular, initially hyaline, becoming brown to blackish when mature, broadly ellipsoid to broadly pip-shaped, truncate with distinct scar at the base, densely multiguttulate, thick-walled, (14–)19–23.5(–28) × (6.5–)9–13(–15) μm (av. = 22 × 12.5 μm, n = 50), with 0.8–1 µm wide gelatinous sheath.

**Figure 4.**
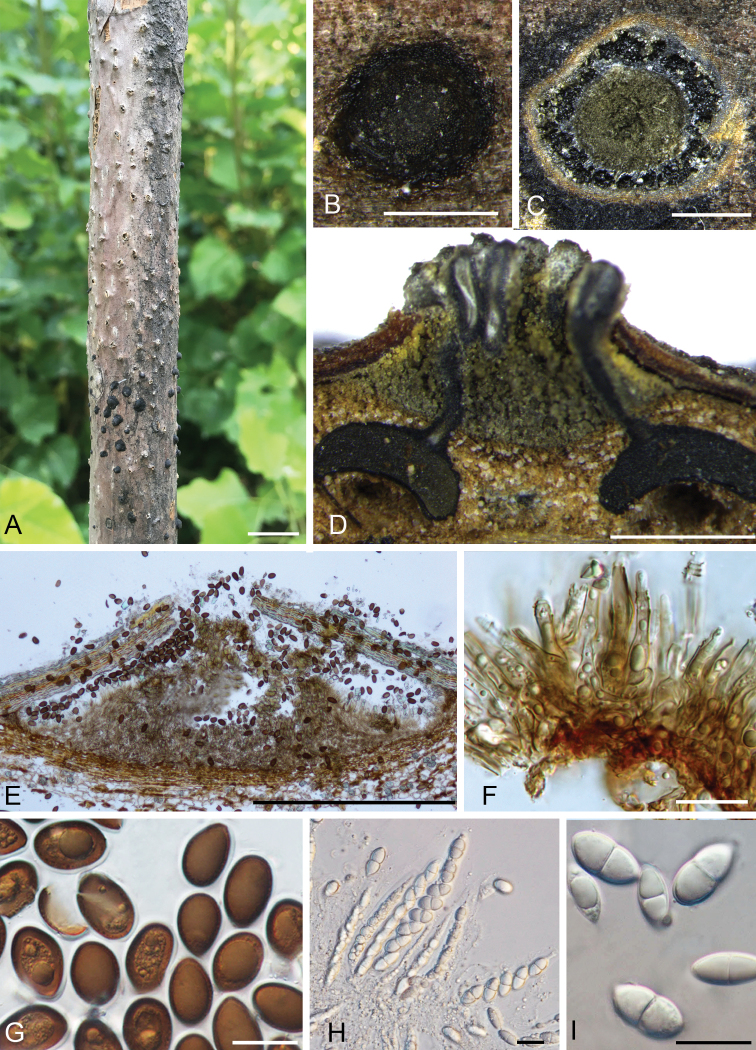
Morphology of *Juglanconisoblonga* from *Juglansregia*. **A–B** habit of acervuli on branches **C** transverse section through acervulus **D** longitudinal section through perithecia **E** longitudinal section through acervulus **F** conidiophores, conidiogenous cells **G** conidia **H** asci and ascospores **I** ascospores. Scale bars: 10 mm (**A**), 500 μm (**B–E**), 20 μm (**F–I**).

#### Culture characteristics.

On PDA, cultures are initially white, becoming pale olivaceous grey after 10 d. The colonies are felty with an irregular edge; texture uniform; sterile.

#### Materials examined.

(all on twigs and branches of *Juglansregia*). CHINA, Heilongjiang Province, Harbin City, Linan, Heilongjiang Botanical Garden, 45°42'21.10"N, 126°38'42.87"E, 2 August 2016, Q. Yang & Z. Du (BJFC-S1374; living culture, CFCC 51725; *ibid.*CFCC 51726).

#### Notes.

*Juglanconisoblonga* is based on *Melanconiumoblongum* (= *Melanconisjuglandis*) (Voglmayr et al. 2017). This species can be distinguished from *J.juglandina* by on average longer length of conidia (22 × 12.5 *vs.>* 20 × 13 µm). However, there is a substantial size overlap between both species and sequence data are sometimes necessary for reliable species identification. It was also recorded to cause canker and dieback disease of *Juglansregia* in China (China Microbiology and Virology Databases, http://www.micro.csdb.cn/).

### Melanconidaceae

Taxon classificationFungiDiaporthalesMelanconidaceae

G. Winter, Rabenh. Krypt.-Fl., Edn 2 (Leipzig) 1.2: 764 (1886)

#### Type genus.

*Melanconis* Tul. & C. Tul., Select. fung. carpol. (Paris) 2: 115 (1863)

#### Notes.

**Figure 5.**
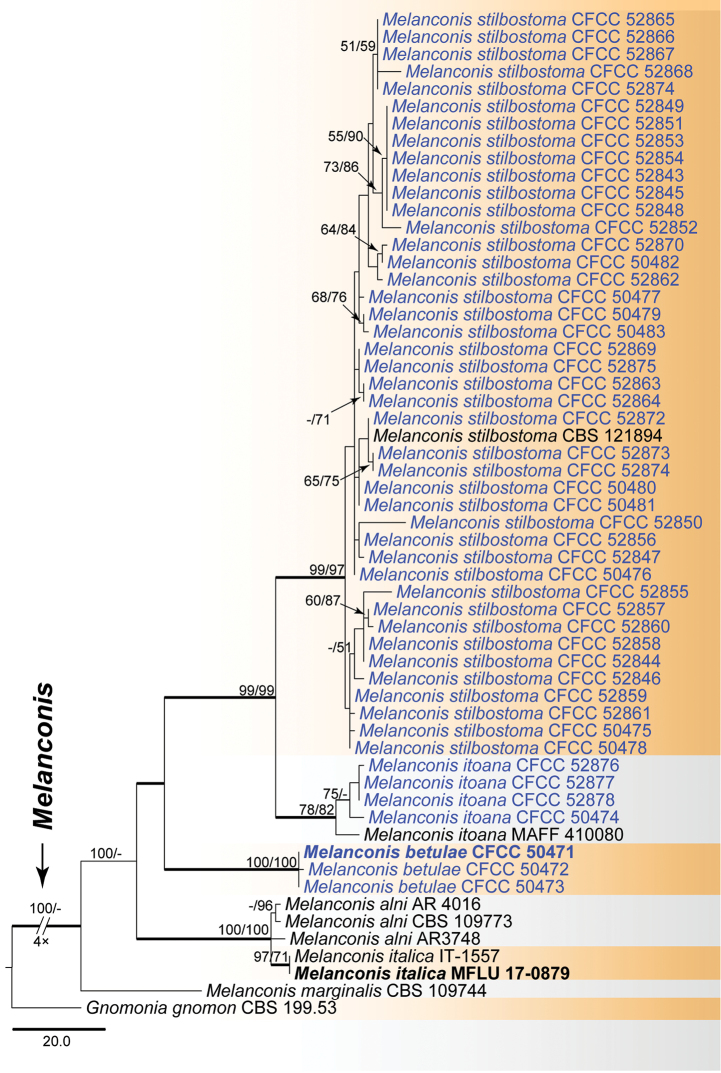
Phylogram of *Melanconis* (Melanconidaceae) obtained from an MP analysis of a combined matrix of ITS, LSU, RPB2 and TEF1-α. MP and ML bootstrap support values above 50% are shown at the first and second position, respectively. Thickened branches represent posterior probabilities above 0.95 from BI. Scale bar = 20 changes. Type species are in bold. Strains obtained in the current study are in blue.

Melanconidaceae was introduced by Winter (1886) and subsequently involved many genera with perithecia immersed in a well-developed stroma with ostioles (beaks) that emerge through an ectostromatic disc (Barr 1978). Castlebury et al. (2002) and Rossman et al. (2007) reduced this family to the type genus *Melanconis* based on LSU rDNA sequences. In this paper, we provide an updated tree with additional isolates of *Melanconis* (Melanconidaceae) from China (Fig. 5). All species have been described and illustrated by Fan et al. (2016).

### Melanconis

Taxon classificationFungiDiaporthalesMelanconidaceae

Tul. & C. Tul., Select. fung. carpol. (Paris) 2: 115 (1863)

#### Type species.

*Melanconisstilbostoma* (Fr.) Tul. & C. Tul., Select. fung. carpol. (Paris) 2: 115 (1863)

#### Notes.

The type genus *Melanconis* was established by Tulasne and Tulasne (1863) based on *Sphaeriastilbostoma* Fr. This genus is characterised by circularly arranged perithecia immersed in well developed to reduced entostromata with a concolourous central column and ostioles erumpent through a light-coloured ectostromatic disc with hyaline, one-septate ascospores; acervuli with light-coloured central column producing brown to olive-brown, fusiform to pyriform alpha conidia and hyaline, cylindrical or allantoid beta conidia (Barr 1978; Castlebury et al. 2002; Voglmayr et al. 2012; Fan et al. 2016). *Melanconis* has approximately 105 species epithets recorded in Index Fungorum (2018), whereas Rossman et al. (2007) suggested that many of the species previously residing in *Melanconis* may belong somewhere else. Fan et al. (2016) provided an account on this genus including five species (*Melanconisalni*, *Ms.betulae*, *Ms.marginalis*, *Ms.itoana* and the type species *Ms.stilbostoma*), which were restricted to hosts in Betulaceae.

### Melanconis betulae

Taxon classificationFungiDiaporthalesMelanconidaceae

C.M. Tian & X.L. Fan, Mycol. Progr. 15(4/40): 4 (2016)

#### Materials examined.

(all on twigs and branches of *Betulaalbosinensis*). CHINA, Gansu Province, Gannan Tibetan Autonomous Prefecture, Zhouqu County, Qiban Forestry Centre, 33°56'35.36"N, 104°07'13.03"E, 20 August 2014, Y.M. Liang (BJFC-S1319, holotype; living ex-type culture, CFCC 50471); Gansu Province, Gannan Tibetan Autonomous Prefecture, Zhouqu County, Qiban Forestry Centre, 33°56'37.05"N, 104°07'13.78"E, 20 August 2014, Y.M. Liang (BJFC-S13200; living culture, CFCC 50472); Gansu Province, Gannan Tibetan Autonomous Prefecture, Zhouqu County, Qiban Forestry Centre, 33°56'34.44"N, 104°07'15.59"E, 20 August 2014, Y.M. Liang (BJFC-S1321; living culture, CFCC 50473).

#### Notes.

*Melanconisbetulae* was described from *Betulaalbosinensis* (Fan et al. 2016). Morphologically, *M.betulae* is characterised by ovoid, olive-brown, aseptate alpha conidia, which are different from other *Melanconis* species but similar to the type species *Ms.stilbostoma*. However, it can be distinguished by the smaller length of its alpha conidia (10 *vs.>* 12 μm) and sequence data.

### Melanconis itoana

Taxon classificationFungiDiaporthalesMelanconidaceae

Tak. Kobay., Bull. Govt Forest Exp. Stn Meguro 226: 19 (1970)

#### Materials examined.

(all on twigs and branches of *Betulaalbosinensis*). CHINA, Gansu Province, Gannan Tibetan Autonomous Prefecture, Zhouqu County, Qiban Forestry Centre, 33°56'34.49"N, 104°07'15.21"E, 20 August 2014, X.L. Fan (BJFC-S1322; living culture, CFCC 50474); Shaanxi Province, Ankang City, Ningshan County, Huoditang Forest Farm, 33°26'24.80"N, 108°26'45.10"E, 3 August 2015, Q. Yang (BJFC-S1349; living culture, CFCC 52877; ibid, CFCC 52878); Jilin Province, Jiaohe City, Haiqing Forest Farm, 43°79'88.71"N, 127°15'83.04"E, 26 June 2017, X.W. Wang (CF 20170668; living culture, CFCC 52876).

#### Notes.

*Melanconisitoana* was described from *Betulaermanii* in Japan (Kobayashi 1970). Fan et al. (2016) isolated it from *Betulaalbosinensis* as a new record in China. *Melanconisitoana* is characterised by fusoid, green-brown alpha conidia with acute ends (13 × 4 μm) and hyaline, cylindrical or crescent beta conidia (9.5 × 1.5 μm).

### Melanconis stilbostoma

Taxon classificationFungiDiaporthalesMelanconidaceae

(Fr.) Tul. & C. Tul., Select. fung. carpol. (Paris) 2: 115 (1863)

#### Materials examined.

(all on twigs and branches of *Betulaplatyphylla*). CHINA, Tibet Autonomous Region, Linzhi City, Juemu Valley, 29°39'50.13"N, 94°18'50.70"E, 22 July 2016, X.L. Fan (CF 20160703; living culture, CFCC 528433); Heilongjiang Province, Yichun City, Dailing District, Liangshui Natural Reserve, 47°11'05.26"N, 128°57'26.15"E, 29 July 2016, Q. Yang & Z. Du (CF 20161703; living culture, CFCC 52867); Heilongjiang Province, Harbin City, Heilongjiang Botanical Garden, 45°42'27.58"N, 126°38'36.72"E, 2 August 2016, Q. Yang & Z. Du (CF 20161709; living culture, CFCC 52868); Qinghai Province, Menyuan City, Xianmi Forest Farm, 37°16'35.27"N, 101°46'53.78"E, 3 September 2016, J.H. Zuo (CF 20160911; living culture, CFCC 52865); Ningxia Autonomous Region, Yinchuan City, Helan County, Taihedizhonghai, 38°31'50.40"N, 106°17'46.10"E, 5 August 2015, X.L. Fan & Z. Du (CF 20150802; living culture, CFCC 52873); Ningxia Autonomous Region, Jingyuan City, Jingguan Road, 35°29'50.32"N, 106°18'27.10"E, 13 August 2014, X.L. Fan & Z. Du (BJFC-S1324; living culture, CFCC 50476); Beijing City, Tongzhou District, Song Village, 35°59'49.50"N, 116°39'32.35"E, 20 May 2015, X.L. Fan (BJFC-S1325; living culture, CFCC 50477); other materials with similar locations and hosts are listed in Table 1.

#### Notes.

*Melanconisstilbostoma* is the type species of *Melanconis* and is thus far only known to occur on *Betula* spp. with a worldwide distribution (Fan et al. 2016). *Betulapendula*, *B.rotundifolia* and *B.tianschanica* are recorded as hosts in China (Zhuang 2005). The current investigation suggested that this species is restricted to and widespread on *Betulaplatyphylla* in China.

### Melanconiellaceae

Taxon classificationFungiDiaporthalesMelanconiellaceae

Senan., Maharachch. & K.D. Hyde, Stud. Mycol. 86: 275 (2017)

#### Type genus.

*Melanconiella* Sacc., Syll. fung. (Abellini) 1: 740 (1882)

#### Notes.

**Figure 6.**
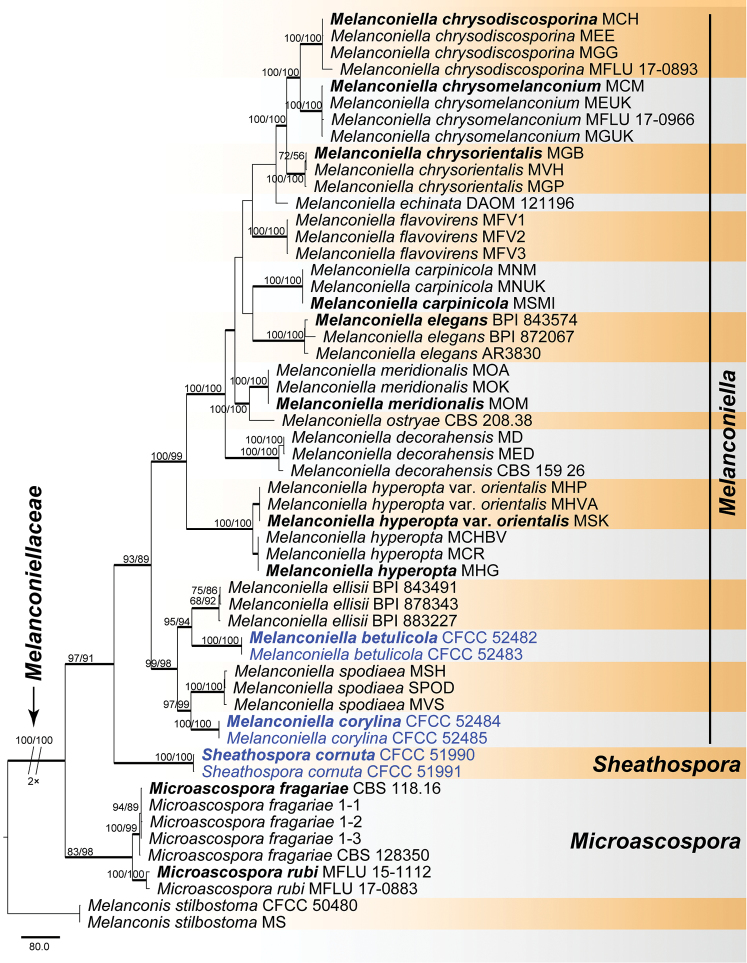
Phylogram of Melanconiellaceae obtained from an MP analysis from a combined matrix of ITS, LSU, RPB2 and TEF1-α. MP and ML bootstrap support values above 50% are shown at the first and second position, respectively. Thickened branches represent posterior probabilities above 0.95 from BI. Scale bar = 80 changes. Type species are in bold. Strains obtained in the current study are in blue.

Melanconiellaceae was validated by Senanayake et al. (2017) for the invalid Melanconiellaceae of Locquin (1984). Senanayake et al. (2017) emended this family to accommodate *Dicarpella*, *Greeneria*, *Melanconiella*, *Microascospora* and *Tubakia*. Braun et al. (2018) recommended an exclusion of *Dicarpella*, *Greeneria* and *Tubakia*. In this paper, we introduce the new genus *Sheathospora* and two new species of *Melanconiella* in Melanconiellaceae (Fig. 6).

### Melanconiella

Taxon classificationFungiDiaporthalesMelanconiellaceae

Sacc., Syll. fung. (Abellini) 1: 740 (1882)

#### Type species.

*Melanconiellaspodiaea* (Tul. & C. Tul.) Sacc., Syll. fung. (Abellini) 1: 740 (1882)

#### Notes.

The genus *Melanconiella* was established by Saccardo (1882) for two species, *Melanconisspodiaea* Tul. & C. Tul. and *M.chrysostroma* (Fr.) Tul. & C. Tul. The genus subsequently entered a long period of confusion with a broad concept of the melanconidaceous genera *Melanconium* and *Melanconis* Tul. & C. Tul. (Wehmeyer 1937, 1941; Barr 1987). *Melanconiella* has 37 species epithets recorded in Index Fungorum (2018). Voglmayr et al. (2012) revised the generic circumscriptions of *Melanconiella* with 13 accepted species, excluded numerous species and confirmed that it is genetically distinct from the genus *Melanconis* based on morphology and multi-gene phylogeny (ITS, LSU, RPB2 and TEF1-α). *Melanconiella* is characterised by forming circularly arranged perithecia immersed in the substrate with oblique or lateral ostioles convergent and erumpent through an ectostromatic disc with dark coloured or hyaline ascospores; acervuli with light-coloured central column, producing dark brown melanconium-like or hyaline discosporina-like conidia (not in the same species) (Barr 1978; Voglmayr et al. 2012). *Melanconiella* species were observed to be highly host-specific, as they were found to be confined to a single genus or sometimes even species within the host family Betulaceae from Europe and North America (Voglmayr et al. 2012).

### Melanconiella betulicola

Taxon classificationFungiDiaporthalesMelanconiellaceae

Fan sp. nov.

828427

Fig. 7

#### Etymology.

*betulicola* (Lat.): referring to the host genus on which it was collected, *Betula*.

#### Diagnosis.

This species is distinguished by hyaline ascospores, (16.5–)18–22(–24) × (3–)4–6 μm, with slightly constricted at the septum and with hyaline broad cap-like appendages at both ends.

#### Holotype.

CHINA. Shaanxi Province: Ningshan County, Huoditang Forest Farm, Huodi Valley, 33°26'36.32"N, 108°26'46.48"E, 3 August 2015, on twigs and branches of *Betulaalbosinensis*, Q. Yang (BJFC-S1347 holotype; living culture, CFCC 52482).

#### Descriptions.

Pseudostromata inconspicuous, immersed in host bark, slightly erumpent from surface of host branches, 1.5–3 mm diam. Ectostromatic disc indistinct, usually circular, buff to hazel. Central column circular, mouse grey to iron grey. Ostioles numerous, violaceous black to black, scarcely projecting, 70–150 μm diam. Perithecia flask-shaped to spherical, arranged circularly or irregularly, 7–12 per disc, often appearing as rounded bumps beneath the bark surface surrounding the ectostromatic disc, (320–)350–550(–610) µm diam. (av. = 480 μm, n = 30). Asci hyaline, clavate to fusoid, (50–)55–65(–70) × (7–)8.5–14(–16) μm (av. = 60 × 11 μm, n = 20). Ascospores hyaline, ellipsoid, broadly ellipsoid or broadly fusoid, 2–4 guttulate, symmetric to slightly asymmetric, straight, rarely slightly curved, slightly constricted at the septum, (16.5–)18–22(–24) × (3–)4–6 μm (av. = 20 × 4.5 μm, n = 50), with hyaline broad cap-like appendages at both ends. Conidiomata acervular, immersed in host bark, erumpent from surface of host branches, scattered or occasionally confluent, 1.3–2.5 mm, covered by fawn to dark brick discharged conidial masses at maturity, usually conspicuous. Ectostromatic disc inconspicuous. Central column beneath the disc more or less conical, olivaceous grey to iron grey. Conidiophores hyaline, smooth, cylindrical to lageniform, simple, rarely branched at the base. Conidiogenous cells hyaline, phialidic. Conidia unicellular, hyaline, narrowly ellipsoid, elongate to slightly allantoid, (9.5–)10–13.5(–15) × (2–)3–4.5(–5.5) μm (av. = 13 × 3.5 µm, n = 50), with 0.5 µm wide gelatinous sheath.

**Figure 7.**
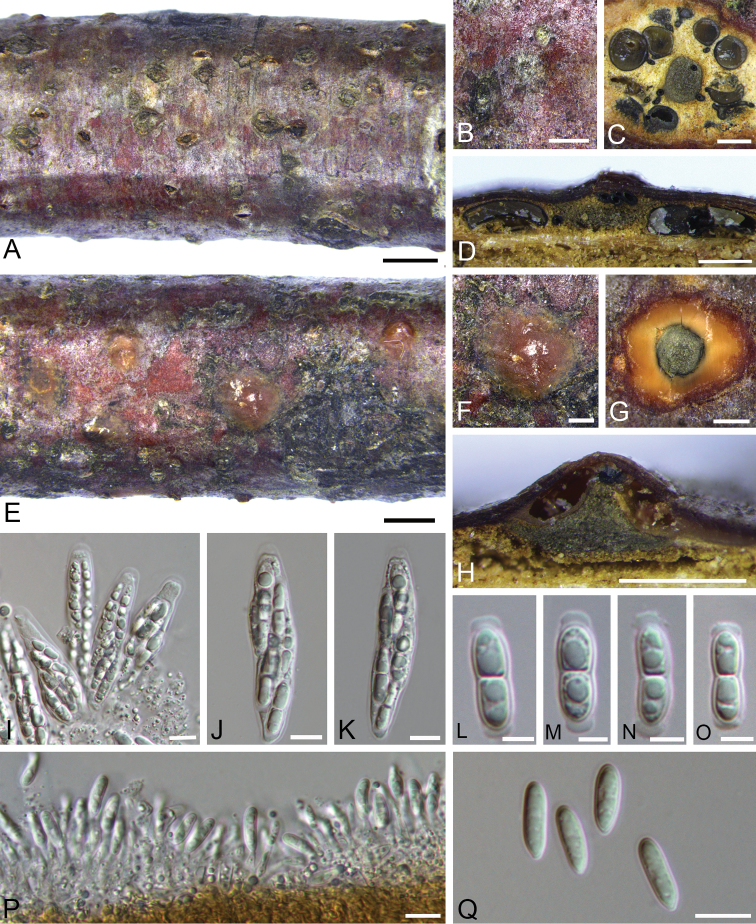
Morphology of *Melanconiellabetulicola* from *Betulaalbosinensis*. **A–B** habit of pseudostromata on branches **C** transverse section through perithecia **D** longitudinal section through perithecia **E–F** habit of acervuli on branches **G** transverse section through acervulus **H** longitudinal section through acervulus **I** asci and ascospores **J–K** ascus and ascospores **L–O** ascospores **P** conidiophores, conidiogenous cells and conidia **Q** conidia. Scale bars: 2 mm (**A, E**), 500 μm (**B–D, F–H**), 10 μm (**J–K, P–Q**), 5 μm (**L–O**).

#### Culture characteristics.

On PDA, cultures are initially white, becoming greyish-sepia after 3 d and distensible radially after 10 d. The colonies are felty with an irregular edge; texture uniform; sterile.

#### Additional material examined.

CHINA. Shaanxi Province: Ningshan County, Huoditang Forest Farm, Huodi Valley, 33°26'37.53"N, 108°26'44.14"E, 3 August 2015, on twigs and branches of *Betulaalbosinensis*, Q. Yang (CF 20150847; living culture, CFCC 52483);

#### Notes.

*Melanconiellabetulicola* is associated with canker disease of *Betulaalbosinensis* in China. It is similar to *M.ellisii* but differs by larger ascospores (18–22 × 4–6 *vs.>* 12.5–16 × 4.0–5.5 μm) with hyaline, broad cap-like appendages at both ends (Voglmayr et al. 2012), distribution (China *vs.>* eastern North America) and a different host, *Betulaalbosinensis vs.> Carpinuscaroliniana*. *Melanconielladecorahensis* also occurs on *Betula* (in Europe and North America) and it can be distinguished from *M.betulicola* based on dark brown ascospores without appendages and dark brown conidia (Voglmayr et al. 2012). The clear phylogenetic position confirmed a distinction from all other available strains included in this study and we therefore result in our decision to describe this species as new, based on DNA sequence data and morphology.

### Melanconiella corylina

Taxon classificationFungiDiaporthalesMelanconiellaceae

Fan sp. nov.

828428

Fig. 8

#### Etymology.

*corylina* (Lat.): referring to the host genus on which it was collected, *Corylus*.

#### Diagnosis.

This species is distinguished by acervuli erumpent through circularly cracked host bark and covered by olivaceous buff to honey discharged conidial masses at maturity; conidia unicellular, hyaline, with various shapes and 1–3 guttulate, (7–)8–13.5(–14.5) × (2–)2.5–4(–5) μm.

**Figure 8.**
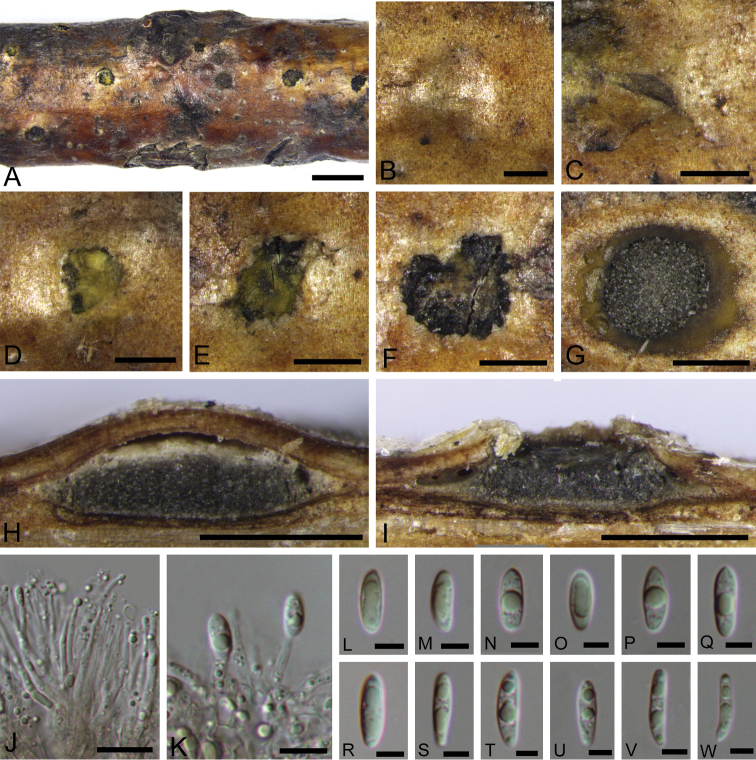
Morphology of *Melanconiellacorylina* from *Corylusmandshurica*. **A** habit of acervuli on branches **B–F** process of development of acervulus **G** transverse section through acervulus **H–I** longitudinal section through acervulus **J** conidiophores **K** conidiogenous cells and conidia **L–W** conidia. Scale bars: 2 mm (**A**), 500 μm (**B–I**), 10 μm (**J–K**), 5 μm (**L–W**).

#### Holotype.

CHINA. Shaanxi Province: Baoji County, Taibai Mountain, 34°15'43.32"N, 107°88'42.16"E, 13 July 2017, on twigs and branches of *Corylusmandshurica*, N. Jiang (BJFC-FB56 holotype; living culture, CFCC 52484).

#### Descriptions.

Conidiomata acervular, immersed in host bark, erumpent from surface of host branches, scattered or occasionally confluent, 1–1.5 mm, erumpent through circularly cracked host bark and covered by olivaceous buff to honey discharged conidial masses at maturity, usually conspicuous. Ectostromatic disc inconspicuous and cracked circularly at maturity. Central column beneath the disc more or less oblate, iron grey to dark grey. Conidiophores hyaline, smooth, cylindrical, simple, rarely branched at the base. Conidiogenous cells hyaline, phialidic. Conidia unicellular, hyaline, narrowly ellipsoid to fusoid, elongate to slightly allantoid, 1–3 guttulate, (7–)8–13.5(–14.5) × (2–)2.5–4(–5) μm (av. = 10 × 3.5 µm, n = 50) μm (av. = 13 × 3.5 µm, n = 50). Sexual morph was not observed.

#### Culture characteristics.

On PDA, cultures are initially white, becoming fuscous black in the centre and edge after 5 d. The colonies are felty with an irregular edge; texture uniform; sterile.

#### Additional material examined.

CHINA. Shaanxi Province: Baoji County, Taibai Mountain, 34°15'40.05"N, 107°88'43.33"E, 13 July 2017, on twigs and branches of *Corylusmandshurica*, N. Jiang (CF 20170756 holotype; living culture, CFCC 52485).

#### Notes.

*Melanconiellacorylina* is associated with canker disease of *Corylusmandshurica* in China. It can be distinguished from its closest relative, the generic type *M.spodiaea* growing in *Carpinus* spp., by its hyaline, discosporina-like conidia, and the smaller size of conidia (8–13.5 × 2.5–4 *vs.>* 13.3–15.2 × 7.5–8.5 μm) as well as the hosts (Voglmayr et al. 2012). *Melanconiellaflavovirens* also occurs on *Corylus* (in Europe and North America), and it can be distinguished from *M.corylina* based on larger conidia (12–15 × 5.0–5.5 *vs.>* 8–13.5 ×2.5–4 μm) (Voglmayr et al. 2012). The phylogenetic inferences indicated *M.corylina* as an individual well-supported clade (MP/ML/BI=100/99/1) within *Melanconiella* and we therefore describe it as new, based on sequence data and morphology.

### Sheathospora

Taxon classificationFungiDiaporthalesMelanconiellaceae

Fan gen. nov.

828429

#### Etymology.

*Sheathospora* (Lat.): referring to the conidia with distinct hyaline sheath.

#### Diagnosis.

This genus differs from other genera in Melanconiellaceae by conical and discrete pycnidia with aseptate, cylindrical to ellipsoidal conidia with distinct hyaline sheath.

#### Type species.

*Sheathosporacornuta* (C.M. Tian & Z. Du) Fan.

#### Descriptions.

Conidiomata pycnidial, immersed in host bark, erumpent through the surface of host branches. Ectostromatic disc inconspicuous and extended to form a beak at maturity. Central column absent. Conidiophores hyaline, smooth, cylindrical, simple, rarely branched at the base. Conidiogenous cells hyaline, phialidic. Conidia hyaline, aseptate, with distinct hyaline sheath. Sexual morph was not observed.

#### Notes.

*Sheathospora* is established for *Melanconiellacornuta*, which was previously included in the *Melanconiella* clade (Voglmayr et al. 2012; Du et al. 2017). Morphologically, it differs from other genera in Melanconiellaceae by pycnidial conidiomata and conidia with distinct hyaline sheath. In our phylogenetic analyses, *Melanconiellacornuta* formed a distinct clade basal to *Melanconiella* within Melanconiellaceae. Based on morphology and different hosts (*Cornus* and *Juglans* vs. Betulaceae), it is here excluded from *Melanconiella* and transferred to the new genus *Sheathospora.* In our revised circumscription, Melanconiellaceae include three genera named *Melanconiella*, *Microascospora* and *Sheathospora*.

### Sheathospora cornuta

Taxon classificationFungiDiaporthalesMelanconiellaceae

(C.M. Tian & Z. Du) Fan comb. nov.

828430

Fig. 9

#### Basionym.

*Melanconiellacornuta* C.M. Tian & Z. Du, Phytotaxa 327(3): 257 (2017)

#### Diagnosis.

This species is distinguished by conical and discrete pycnidia without central column and aseptate, cylindrical to ellipsoidal, (19–)19.5–22.5(–23) × (8–)8.5–10.5(–11) μm conidia, with a distinct hyaline sheath 1–1.5 μm wide.

#### Holotype.

CHINA. Shaanxi Province: Ankang City, Ningshan County, Huoditang Forest Farm, 33°26'04.46"N, 108°26'59.91"E, 3 July 2016, on twigs and branches of *Cornuscontroversa*, X.L. Fan (BJFC-S1375 holotype; living ex-type culture CFCC 51990).

#### Descriptions.

Conidiomata pycnidial, immersed in host bark, conical, with single necks erumpent through the surface of host branches, scattered, (250–)270–330(–410) μm (av. = 300 μm, n = 20) diam. Ectostromatic disc inconspicuous and extended to form a beak at maturity, pale luteous to amber. Central column absent. Conidiophores hyaline, smooth, cylindrical, simple, rarely branched at the base, 17–24(–25) × 2.5–4(–4.5) μm (av. = 21.5 × 3.5 µm, n = 50). Conidiogenous cells hyaline, phialidic. Conidia hyaline, aseptate, cylindrical to ellipsoidal, (19–)19.5–22.5(–23) × (8–)8.5–10.5(–11) μm (av. = 21 × 10 µm, n = 50), with distinct hyaline sheath, 1–1.5 μm wide at maturity. Sexual morph was not observed.

**Figure 9.**
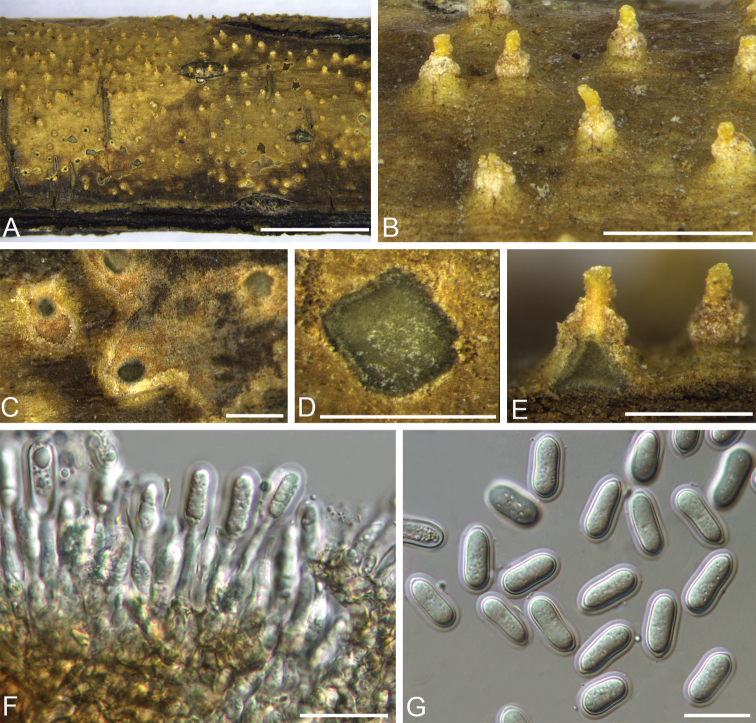
Morphology of *Sheathosporacornuta* from *Cornuscontroversa*. **A–B** Habit of pycnidia on branches **C–D** transverse section through pycnidium **E** longitudinal section through pycnidium **F** conidiophores, conidiogenous cells **G** conidia. Scale bars: 5 mm (**A**), 1 mm (**B**), 500 μm (**C–E**), 20 μm (**F–G**).

#### Culture characteristics.

Colony growth on PDA originally white, becoming pale yellowish after 7–10 days. Colony flat, felty-like, with a uniform texture and yellowish to dark brown conidiomata irregularly scattered on the colony surface.

#### Additional specimens examined (paratypes).

CHINA. Shaanxi Province: Ankang City, Ningshan County, Huoditang Forest Farm, 36°26'13.30"N, 108°26'48.32"E, 3 August 2015, on twigs and branches of *Juglansregia*, Q. Yang (BJFC-S1345 paratype; living ex-paratype culture CFCC 51991).

#### Notes.

*Sheathosporacornuta* is proposed here as a new combination for *Melanconiellacornuta*. It is the type and currently only species of *Sheathospora* and so far known from *Cornuscontroversa* and *Juglansregia* in China. The sexual morph of this species is unknown and further collections are required to elucidate its life cycle.

## Discussion

During the investigation of melanconis-like fungi in China, we identified eight species residing in three families (Juglanconidaceae, Melanconidaceae and Melanconiellaceae) of Diaporthales. It includes *Juglanconisjuglandina*, *J.oblonga*, *Melanconisbetulae*, *Ms.itoana*, *Ms.stilbostoma*, the two new species *Melanconiellabetulicola* and *M.corylina* and the new combination *Sheathosporacornuta* in the new genus *Sheathospora*.

All specimens in the current study were collected from symptomatic branches and twigs associated with canker or dieback diseases, of which *Juglanconis* (Juglanconidaceae) species were isolated from *Juglansregia* (Juglandaceae), *Melanconiella* (Melanconiellaceae) species from *Betulaalbosinensis* and *Corylusmandshurica* (Betulaceae) and *Melanconis* (Melanconidaceae) species from *Betulaalbosinensis* and *Betulaplatyphylla* (Betulaceae). It may indicate that many melanconis-like species have obvious host specificity. The type species of the new genus *Sheathospora* (Melanconiellaceae) was isolated from Cornaceae (*Cornuscontroversa*) and *Juglansregia* (Juglandaceae), suggesting a low host specificity and that additional undiscovered hosts species of this taxon may exist in China.

As the morphological features in previous melanconis-like fungi are highly overlapping, phylogenetic studies using DNA sequences have been useful to elucidate the diversity and systematics in this group. The current results indicated that *Juglanconis* and *Melanconis* are still unique, the only genera in Juglanconidaceae and Melanconidaceae, respectively, due to the lacking of extensive fresh collections. The family Melanconiellaceae was recently proposed by Senanayake et al. (2017) to accommodate *Dicarpella*, *Greeneria*, *Melanconiella*, *Microascospora* and *Tubakia* based on morphological features and phylogenetic analyses. In this study, the phylogenetic affinity of *Dicarpella*, *Greeneria* and *Tubakia* was evaluated in Diaporthales (Fig. 1), which conformed to the recently described family Tubakiaceae (Diaporthales) (Braun et al. 2018). We here establish a new genus within Melanconiellaceae, *Sheathospora*, which is characterised by typical diaporthalean-like pycnidia and aseptate, cylindrical to ellipsoidal conidia with distinct hyaline sheath. Thus Melanconiellaceae is here restricted to the three genera *Melanconiella*, *Microascospora* and *Sheathospora* (Fig. 6).

As shown in this paper, future studies addressing the fungal diversity associated with canker or dieback diseases should routinely include sequence data for protein-coding genes to achieve stable, supported topologies in phylogenetic trees. It is hoped that the classification proposed here will also provide an updated phylogenetic framework that will facilitate further revision of the families with melanconis-like asexual morphs. Although the current study provides additional new data on melanconis-like genera, typification, species concept and taxonomic affiliation of many described *Melanconium* species are yet unclear, including the type species *M.atrum*, which currently represents a doubtful taxon (Rossman et al. 2015). In addition, sequence data are missing for most described *Melanconium* species. Thus, a thorough revision of the genus *Melanconium* based on robust sampling, reliable identification, cultures and DNA data is urgently needed. The fact that new records and species from three related families of Diaporthales were recorded in China further suggests that Asia may harbour many more species awaiting collections and descriptions.

## Supplementary Material

XML Treatment for Juglanconidaceae

XML Treatment for Juglanconis

XML Treatment for Juglanconis juglandina

XML Treatment for Juglanconis oblonga

XML Treatment for Melanconidaceae

XML Treatment for Melanconis

XML Treatment for Melanconis betulae

XML Treatment for Melanconis itoana

XML Treatment for Melanconis stilbostoma

XML Treatment for Melanconiellaceae

XML Treatment for Melanconiella

XML Treatment for Melanconiella betulicola

XML Treatment for Melanconiella corylina

XML Treatment for Sheathospora

XML Treatment for Sheathospora cornuta
